# Towards a unified understanding of the copper sites in particulate methane monooxygenase: an X-ray absorption spectroscopic investigation†

**DOI:** 10.1039/d1sc00676b

**Published:** 2021-03-30

**Authors:** George E. Cutsail, Matthew O. Ross, Amy C. Rosenzweig, Serena DeBeer

**Affiliations:** Max Planck Institute for Chemical Energy Conversion Stiftstrasse 34-36 D-45470 Mülheim an der Ruhr Germany george.cutsail@cec.mpg.de serena.debeer@cec.mpg.de; University of Duisburg-Essen Universitätsstrasse 7 D-45151 Essen Germany; Departments of Molecular Biosciences and Chemistry, Northwestern University Evanston 60208 IL USA

## Abstract

The enzymatic conversion of the greenhouse gas, methane, to a liquid fuel, methanol, is performed by methane monooxygenases (MMOs) under mild conditions. The copper stoichiometry of particulate MMO (pMMO) has been long debated, with a dicopper site previously proposed on the basis of a 2.51 Å Cu–Cu feature in extended X-ray absorption fine structure (EXAFS) data. However, recent crystallographic data and advanced electron paramagnetic resonance (EPR) characterization support the presence of only mononuclear copper sites. To reconcile these data, we have collected high-energy resolution fluorescence detected (HERFD) and partial fluorescence yield (PFY) EXAFS spectra of *Methylococcus* (*M*.) *capsulatus* (Bath) pMMO. Both methods reveal only monocopper sites. These data were compared to previously published pMMO PFY-EXAFS data from *M. capsulatus* (Bath) and *Methylomicrobium alcaliphilum* 20Z, supporting dicopper and monocopper sites, respectively. The FT-EXAFS feature previously attributed to a dicopper site can be reproduced by the inclusion of a metallic copper background signal. The exact position of this feature is dependent on the nature of the sample and the percentage of background contamination, indicating that visual inspection is not sufficient for identifying background metallic contributions. Additionally, an undamaged X-ray absorption spectrum was obtained, consistent with the copper oxidation-state speciation determined by EPR quantification. X-ray photodamage studies suggest that the previously observed Cu(i) XAS features are in part attributable to photodamage. This study illustrates the complex array of factors involved in EXAFS measurement and modeling of pMMO and more generally, dilute metalloproteins with multiple metal centers.

## Abstract 2

Extended X-ray absorption fine structure spectroscopic analysis of particulate methane monooxygenase reveals only monocopper sites and investigates the possible origins of the previous observed dicopper signals.

## Graphical abstract

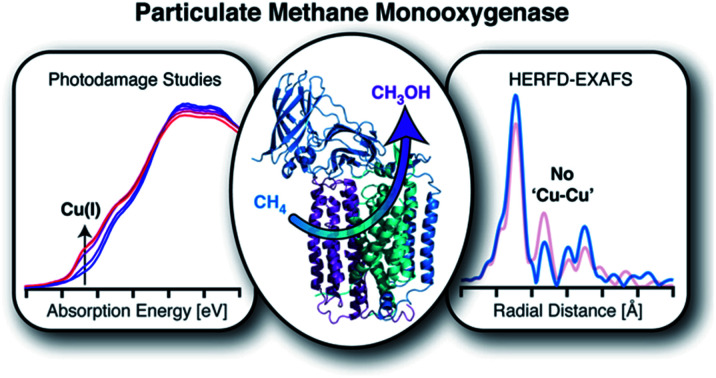

## Introduction

**Fig. 1:**
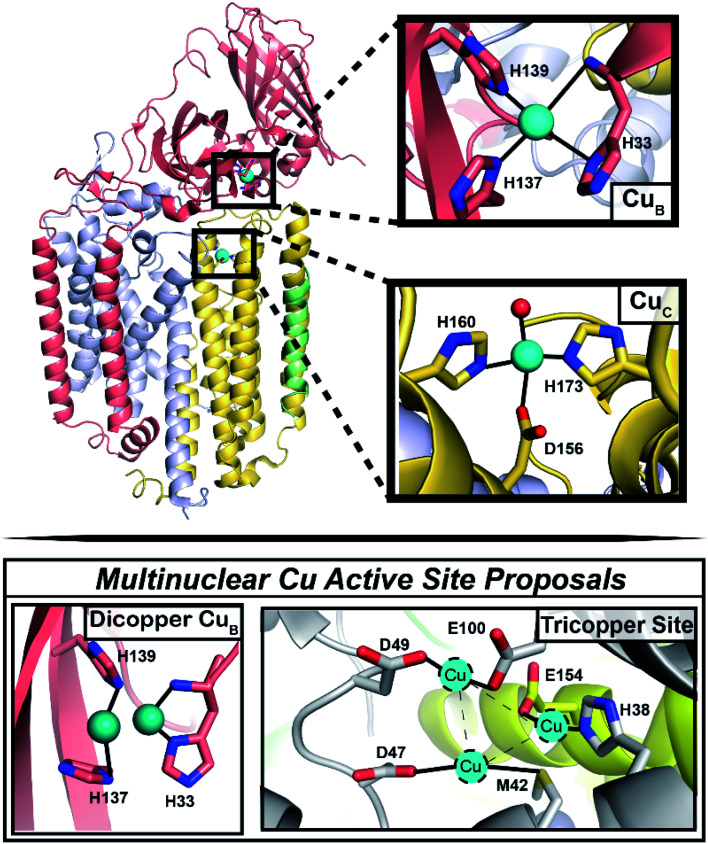
Architecture of the pMMO protomer, with focus on the Cu_B_ and Cu_C_ sites. (Top) Shown is one protomer (the pMMO trimer comprises three PmoB/PmoA/PmoC protomers) of the Rockwell-pMMO crystal structure (PDB: 4PHZ)^20^ with *M. capsulatus* (Bath) residue numbering and PmoA in gray, PmoB in salmon, PmoC in yellow, and an unidentified helix in green. Cu atoms are teal spheres while the red sphere corresponds to the O from an H_*x*_O molecule. (Bottom) The two previous proposals for a multinuclear copper active site.^9,11–13^

Selective partial oxidation of methane to methanol offers an attractive route towards valorization of this abundant, underutilized gas. However, industrial processes for effecting the conversion of methane to methanol require extreme temperature and pressure, as well as significant capital investment in infrastructure, rendering such processes economically inviable for much of the vast natural methane deposits.^1–4^ Alternatively, methanotrophic bacteria^5^ have evolved two genetically unrelated enzymes to catalyze methane to methanol conversion under ambient conditions: soluble and particulate methane monooxygenase (sMMO and pMMO, respectively).^6^ sMMO features a well-characterized diiron active site,^7,8^ and while pMMO is generally accepted as a copper enzyme,^6,9,10^ the identity of its catalytic copper cofactor has proved far more elusive, severely impeding our mechanistic understanding of this important enzyme.^11^ Much of the literature over the past two decades has focused on the possibility of a multinuclear copper active site, with the main proposals involving a tricopper site in the PmoA subunit and a dicopper site in the PmoB subunit (Fig. 1).^9,11–13^ While a metal binding site in PmoA has never been observed crystallographically, crystal structures of pMMOs isolated from multiple methanotrophs all contain copper in PmoB (Cu_B_ site), modeled as either dicopper or monocopper.^6^ Defining the nuclearity and ligation of the pMMO copper cofactors is a fundamental step towards elucidating a catalytic mechanism.

**Fig. 2:**
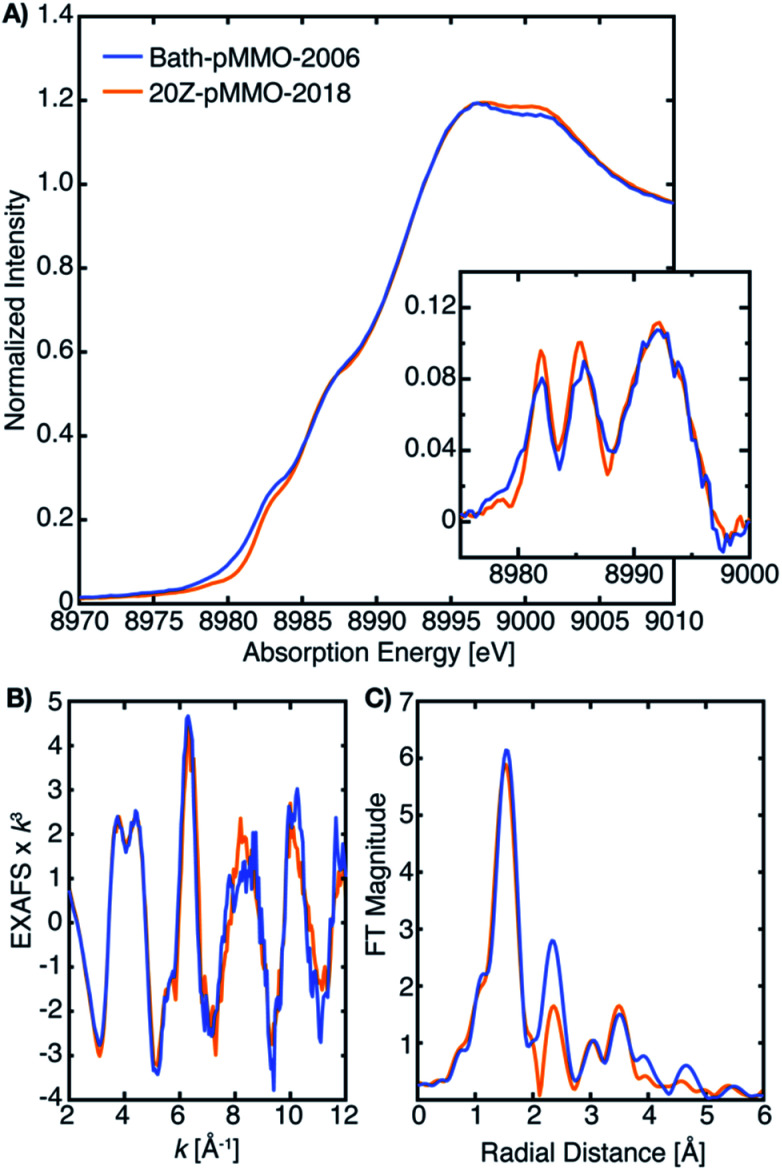
(A) Partial fluorescent yield (PFY) Cu-XAS of **Bath-pMMO-2006** and **20Z-pMMO-2018**. The first derivative of the edge region is shown in the inset of panel (A). (B) Raw *k*^3^-weighted EXAFS and (C) the non-phase shifted Fourier transform taken over a *k*-range of 2 to 12 Å^−1^. Data for **Bath-pMMO-2006** adapted with permission from ref. 16. Copyright 2006 American Chemical Society. Data for **20Z-pMMO-2018** adapted from ref. 23. This research was originally published in the Journal of Biological Chemistry. Copyright the American Society for *Biochemistry and Molecular Biology*.

The Cu_B_ site was first modeled as dicopper in the *Methylococcus* (*M.*) *capsulatus* (Bath) pMMO (Bath-pMMO) crystal structure (Fig. 1).^14^ Although the two copper ions were not individually resolved in the 2.8 Å resolution structure,^14^ a ∼2.5 Å Cu–Cu scattering interaction was first modeled in the extended X-ray absorption fine structure (EXAFS) data for a feature observed at *R* ∼2.3 Å in the Fourier transform, Fig. 2C,^15,16^ prompting modeling the electron density as a dicopper center.^14^ Subsequent quantum refinement of the Bath-pMMO structure supported a mononuclear Cu_B_ site, however.^17^ Short Cu–Cu scattering interactions were also modeled from the EXAFS data for pMMOs from *Methylosinus trichosporium* OB3b,^18^*Methylocystis* species (sp.) strain M,^19^ and *Methylocystis* sp. strain Rockwell (Rockwell-pMMO),^20^ although the electron density for these structures was overall well fit with a single copper ion.

The physiological relevance of the crystallographically-observed copper sites was recently investigated through advanced paramagnetic resonance studies of whole cell and purified Bath-pMMO samples.^21^ These data revealed the presence of two monocopper centers (Fig. 1), the first assigned to the Cu_B_ site on the basis of its nitrogen hyperfine couplings and the second assigned in accordance with double electron–electron resonance (DEER) spectroscopic data to a location in the PmoC subunit (Cu_C_) that can be occupied by zinc (a crystallographic artifact)^14,19^ or copper^18,20^ in crystal structures. A native top-down mass spectrometry study of *Methylomicrobium alcaliphilum* 20Z pMMO (20Z-pMMO) and Rockwell-pMMO also indicated that the PmoB and PmoC subunits each contain a single monocopper binding site.^22^ Importantly, this work further demonstrated that Rockwell-pMMO reconstituted into nanodiscs with copper supplementation has both greater occupancy of one copper equivalent bound to the PmoC subunit and increased methane oxidation activity. This finding is consistent with the notion that the Cu_C_ site may be the catalytic active site instead of the previously suggested Cu_B_ site.^21^

The most recent EXAFS study of pMMO using 20Z-pMMO (**20Z-pMMO-2018**) showed no evidence for a short Cu–Cu scattering contribution,^23^ in contrast to all previously reported pMMO Cu EXAFS data. The observed differences were attributed to various possible scattering interactions of the sample or the presence of other contaminant copper proteins that deconstructively cancel the Cu–Cu features. Curiously, the published Cu K-edge X-ray absorption spectra (XAS) of purified Bath-pMMO^16^ (**Bath-pMMO-2006**) and **20Z-pMMO-2018** (ref. 23) are nearly superimposable (Fig. 2), despite differences in copper oxidation state distributions measured by electron paramagnetic resonance (EPR) spin quantitation, with Bath-pMMO containing ∼40% Cu(ii) and 20Z-pMMO containing ∼85% Cu(ii).^16,23^ The Cu K-edge XAS data shown in Fig. 2 are similar to all reported spectra for purified pMMOs. As the Cu K-edge is very sensitive to metal oxidation state, the similarities of the two Cu K-edge spectra do not match the copper oxidation state distribution determined by EPR spectroscopy.

As exemplified by the 20Z-pMMO EXAFS study and despite the increasing evidence that pMMO contains only mononuclear copper sites, researchers in this field have been unable to account for the fact that EXAFS analyses of multiple pMMO samples and recombinant constructs, performed by multiple investigators working independently, consistently measured a short distance (∼2.5–2.7 Å) Cu–Cu scattering interaction.^15,16,18–20,24–27^ In order to advance the mechanistic understanding of the enzyme, the previous EXAFS studies must collectively be reconciled with themselves and with the growing body of experimental evidence indicating exclusively monocopper centers in pMMO. Herein, we first revisit the Cu XAS of pMMO to understand how various samples could have very similar XAS spectra and yet different copper oxidation state distributions based on EPR. Secondly, in our efforts to study the origins of the previously assigned Cu–Cu scattering interaction of Bath-pMMO, we have collected both high-energy resolution fluorescence detected (HERFD) and partial fluorescence yield (PFY) EXAFS, the latter of which was employed in all prior pMMO EXAFS studies. We have previously demonstrated for the diiron sMMO protein that the optical configuration of the Bragg crystal spectrometer employed in HERFD-EXAFS eliminates background metallic contributions.^28^ Hence, in the current study, the use of HERFD-EXAFS allows us to evaluate the possibility of background copper metallic scattering contributions to the originally published EXAFS data. We have also modeled the newly collected EXAFS of Bath-pMMO (in both PFY and HERFD detection modes) and remodeled the previously published EXAFS of Bath-pMMO^16^ and 20Z-pMMO.^23^ These new data sets, evaluated in parallel with previously published data, allow for a more holistic understanding of the XAS and EXAFS of pMMO.

## Results and discussion

To reinvestigate the XAS and EXAFS of pMMO, two batches of purified Bath-pMMO were prepared for XAS analysis, one highly concentrated set of samples (833 μM pMMO protomer, 2.0 mM total Cu) and the other at a lower concentration (256 μM protomer, 0.61 mM total Cu). These samples are referred to as **Bath-pMMO-2021** (high/low conc.) in which high or low indicates the relative protein concentration. Characterization of **Bath-pMMO-2021** samples by X-band EPR spectroscopy revealed that both samples exhibited the expected Cu_B_(ii) and Cu_C_(ii) EPR signatures, as measured in previous studies (Fig. S1†).^21^ These samples contained 2.4 ± 0.14 Cu per protomer as measured by inductively coupled plasma-optical emission spectroscopy (ICP-OES), of which 71–83% is Cu(ii) as determined by EPR spin quantitation (Table 1). The newly collected data are compared throughout this work to the original XAS and EXAFS data for **Bath-pMMO-2006** (1 mM total Cu).^16^ The XAS and EXAFS of **20Z-pMMO-2018** (ref. 23) (385 μM protomer; ∼1.0 mM total Cu) are also included for comparisons.

**Table 1:** Copper quantification and oxidation-state distribution

Sample	Total Cu^a^	Cu(ii)^a^^,^^b^	% Cu(ii)^b^	Normalized intensity at 8983.0 eV^c^
Bath-pMMO-2021 (high conc.)	2.4	1.7	71	0.096
Bath-pMMO-2021 (low conc.)	2.4	2.0	83	0.113
Bath-pMMO-2021 (low conc.) damaged	2.4	—	—	0.265
20Z-pMMO-2018^d^	2.7	2.3	∼85	0.247
Bath-pMMO-2006^e^	2–4	0.8–1.6	∼40^e^	0.273

a Per protomer.

b Quantified by EPR spectroscopy prior to XAS measurements.

c Normalized intensities of Cu K-edge XAS at 8983.0 eV determined from Fig. 3. Photodamage studies of **Bath-pMMO-2021** show that this feature is the result of photodamage of Cu(ii) to Cu(i) and not native Cu(i).

d Quantifications from Ro *et al.*^23^

e Original Cu(ii) estimates of pMMO-Bath-2006 were 40%,^16^ corresponding to the XAS reproduced here. An earlier study^15^ estimated 40–60% Cu(ii) and yielded a similar Cu XAS spectrum. Approximately ∼25–50% Cu(ii) has been more recently quantified,^23,29^ but no XAS studies were performed on those samples.

### X-ray absorption spectra of pMMO

As with most Cu(ii)-containing samples, the **Bath-pMMO-2021** samples, containing a majority of Cu(ii), are sensitive to photodamage by the intense synchrotron radiation used. Consecutive scans of the Cu K-edge measured by PFY detection allow for photodamage to the sample to be monitored. Initial scans of both the high and low concentration samples under very low-flux conditions showed a pre-edge feature at 8978 eV characteristic of a 1s → 3d transition into the single d-hole of a Cu(ii) center, Fig. 3. The prominent 8983 eV feature assigned to a Cu(i) 1s → 4p transition in previously published pMMO spectra^16,23^ is not present. Instead, a broad, weak shoulder is observed at 8983 eV that is more easily recognized in the derivative spectrum (Fig. 3, inset). In addition, the white-line features at ∼8997 to 9003 eV of **Bath-pMMO-2021** recorded here are at slightly higher energy than previously reported. The observed copper XAS for the **Bath-pMMO-2021** samples is consistent with a majority Cu(ii) oxidation speciation, as also determined by EPR. Although the copper speciation (stoichiometry and oxidation states) of **Bath-pMMO-2021** measured here and of **20Z-pMMO-2018** measured previously are nearly the same as determined by EPR and ICP-OES, their Cu XAS spectra differ. In particular, the **20Z-pMMO-2018** 8983 eV feature is not consistent with the copper oxidation state speciation determined by EPR (Fig. 3 and Table 1).

**Fig. 3:**
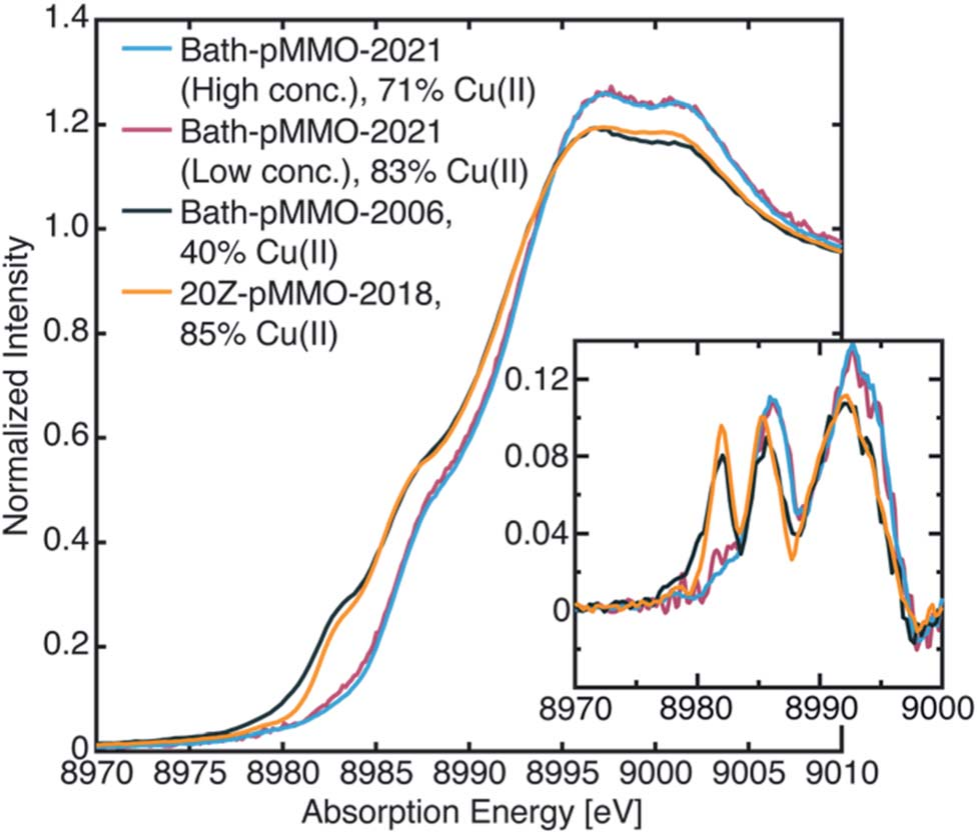
Newly collected Cu PFY-XAS of **Bath-pMMO-2021** compared with previously published XAS of **Bath-pMMO-2006** (ref. 16) and **20Z-pMMO-2018**.^23^ The percent Cu(ii) speciation is from EPR spectroscopic quantification as detailed in Table 1. Inset depicts first derivative spectra.

### Effects of photodamage on pMMO XAS and EXAFS

**Fig. 4:**
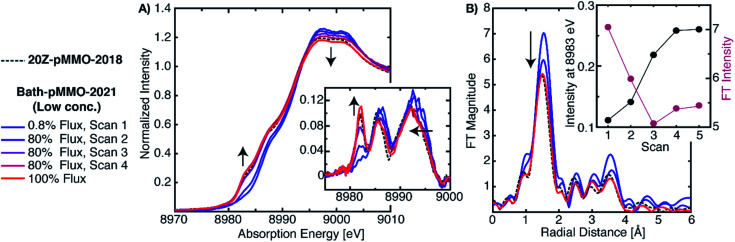
(A) Normalized PFY-XAS spectra of **Bath-pMMO-2021** acquired at various photodamage levels. The low-dose (0.8%) scan corresponds to the average of several fresh sample spots to achieve adequate signal-to-noise for EXAFS analysis. The higher flux scans were subsequently taken at a single previously exposed 0.8% flux sample spot. All scans exposed the samples for approximately 20 min. The inset of A shows the first derivative spectra over a narrower energy range. Arrows indicate observed trends as a function of photodamage. The data are presented with the normalized XAS spectrum of **20Z-pMMO-2018**.^23^ (B) Non-phase shifted EXAFS Fourier transform (FT) of **Bath-pMMO-2021**. All FTs, including those of **20Z-pMMO-2018**, were calculated over a *k*-range of 2–10 Å^−1^ from the unfiltered *k*^3^-weighted EXAFS spectra. The inset of B shows the inverse correlation of the 8983 eV feature intensity with the intensity of the first radial shell of the FT-EXAFS spectra.

To address the possible origins of this discrepancy, we performed extensive radiation studies to reduce and mitigate photodamage. The Cu XAS spectra of **Bath-pMMO-2021** in Fig. 3 were obtained with less than 1.0% of the available flux at wiggler beam line 9–3 at Stanford Synchrotron Radiation Laboratory (SSRL). Consecutive EXAFS scans reveal the growth of the Cu(i) feature at 8983 eV, Fig. 4, S2 and S3.† This feature appears to saturate or reach maximum intensity after four 20 minute scans, and the white-line feature has shifted to lower energy after photodamage, as clearly observed in the derivative spectra. Interestingly, using the full flux on the same spot does not result in significant further damage relative to scan 4. The saturation of the damage is most clearly demonstrated by plotting the intensity of the 8983 eV feature *versus* exposure time, as shown in the insets of Fig. 4A and B. The rise of the 8983 eV feature appears nearly instantaneously as measured during fixed incident energy time scans, Fig. S4.† This observation provides an important cautionary note that damage effects can become saturated quickly, and without significant attenuation of the beam, may be missed entirely. In this context, we note that the photodamaged spectrum of **Bath-pMMO-2021** measured here matches the previously published spectrum of **20Z-pMMO-2018** (Fig. 3).

Although the 8983 eV feature has been used as a marker for the presence of Cu(i), it is also well known that the energy and intensity of Cu(i) 1s → 4p transitions in this region are sensitive to both geometry and coordination number, particularly when examining related ligand sets.^30^ While the influence of photodamage on the pMMO XAS spectrum is not fully understood, there are notable similarities between the XAS spectra of Bath-pMMO and Cu(ii)-coordinated amyloid-β peptide (Cu:Aβ), Fig. S5.† The average coordination spheres of pMMO and Cu(ii):Aβ are similar, with a mixture of N and O ligands.^31–34^ Previous radiation damage studies of Cu(ii):Aβ exhibit the growth of a Cu(i) feature at 8983 eV (ref. 32) identical to that observed for damaged **Bath-pMMO-2021** (Fig. S5†), and similar to the **Bath-pMMO-2006** and **20Z-pMMO-2018** spectra (Fig. 3 and 4). The photodamage studies of Cu(ii):Aβ also demonstrate that the photodamage Cu(i) product is not the same as the Cu(i) observed by chemical reduction.^32,35^ Similarly, slight differences between the chemically-reduced spectrum of **Bath-pMMO-2006** and that of damaged **Bath-pMMO-2021** are observed, Fig. S5.†

While the discrepancy between the similar Cu(ii) EPR quantifications (Table 1) and the different XAS spectra (Fig. 3) for **Bath-pMMO-2021** and **20Z-pMMO-2018** may be reconciled by a photodamage hypothesis for **20Z-pMMO-2018**, a photodamage-only mechanism cannot entirely explain the XAS spectrum of **Bath-pMMO-2006**, which contained a higher percentage of Cu(i) (Table 1). Both the Cu(i) initially present in the sample (presumably natively reduced protein) and Cu(i) resulting from photodamage may contribute to the intensity of the 8983 eV of **Bath-pMMO-2006**, underscoring the necessity to mitigate photodamage in order to observe the species of interest. The presence of a photodamaged Cu(i) state precludes full characterization the native Cu(i) species present in any given sample.

Photodamage also influences the EXAFS of pMMO. Consecutive EXAFS scans on increasingly photodamaged samples yielded moderately different FT-EXAFS spectra (Fig. 4B). The FT of the minimally damaged (scan 1) sample has the most intense radial shell at *R* ∼1.65 Å of approximately 7 units of intensity. An immediate decrease of this radial shell is observed in the second scan at the same sample spot, showing that photodamage influences the first coordination sphere. Since this shell corresponds to the sum of the first coordination shell scattering interactions, photodamage to the copper center and subsequent, but unknown, alterations in coordination will influence its intensity. If the photodamaged product has different mean bond lengths than the undamaged copper center, the EXAFS signal will be an average of all the undamaged and damaged scattering interactions, and this increase in static disorder, modeled as the e^−2*σ*2*k*2^ portion of the EXAFS equation, will decrease the observed intensity of the feature. Consistent with the saturation of the XAS 8983 eV feature after the third scan, the FT intensity of the radial shell at *R* ∼1.65 Å decreases significantly in the first two scans and then appears to reach a level close to saturation (Fig. 4B). The photodamaged EXAFS of **Bath-pMMO-2021** matches that of **20Z-pMMO-2018**, Fig. 4B, with similar first shell radial intensities (∼5.5 units). Both EXAFS data sets were collected on the same beamline and reprocessed together over a *k*-range of 2 to 10 Å^−1^. Given the similarities in XAS and EXAFS spectra, and the approximately equal amounts of Cu(ii), it is likely that the previously published **20Z-pMMO-2018** data represent a photodamaged sample.^23^

### EXAFS of pMMO

The previously proposed dicopper Cu_B_ site was supported primarily by the short Cu–Cu scattering interaction observed in the FT of the EXAFS spectrum at *R* ∼2.3 Å (non-phase shifted), Fig. 2. The Cu–Cu scattering interaction of **Bath-pMMO-2006** was modeled with a coordination number of *N* = 0.25. This is significantly lower than the ideal 0.66 expected for the average of three coppers (*i.e.* a dicopper Cu_B_ and a monocopper Cu_C_) or 0.50 if the mononuclear bis-His Cu site observed in the Bath-pMMO crystal structure^14^ is included. Additionally, the Debye–Waller value of the Cu–Cu scattering path is somewhat large (4.65 × 10^−3^ Å^2^), reflecting a high degree of disorder for the modeled Cu–Cu interaction. The combined low coordination number and larger Debye–Waller value do not support a well-formed, fully occupied dicopper site. While purification and sample preparation could disrupt a dicopper site, recent studies indicate that pMMO only contains monocopper sites,^21,22^ suggesting that this feature might have derived from other scattering interactions, including contaminant contributions, photodamage effects, or more complex constructive/deconstructive scattering interactions.

The Cu–Cu scattering feature of **Bath-pMMO-2006** (ref. 16) is not observed in the EXAFS of **20Z-pMMO-2018** (ref. 23) nor **Bath-pMMO-2021**, although it was observed for Rockwell-pMMO^20^ along with pMMOs from *Methylosinus trichosporium* OB3b^18^ and *Methylocystis* sp. strain M.^19^ Photodamage experiments of **Bath-pMMO-2021** show that increasing exposure influences the FT-EXAFS spectrum by decreasing the intensity of the first radial shell, Fig. 4B. Additionally, a decrease in the intensity of the histidine multiple-scattering paths at *R* ∼3.6 Å is observed with continued exposure, Fig. 4B, potentially suggesting damage to coordinated histidine ligands. Continued X-ray exposure, however, does not yield intensity at *R* ∼2.3 Å in the FT-EXAFS spectra indicating that photodamage is not a plausible mechanism to generate the previously observed Cu–Cu scattering interaction, and other possible contributions must be considered.

### HERFD- *vs.* PFY-EXAFS collection

Previously, we evaluated the diiron site in sMMO by both HERFD- and PFY-XAS collection techniques.^28^ This work demonstrated that standard PFY-EXAFS data collection on dilute metalloproteins is more prone to contamination by metallic background signals, while the HERFD-EXAFS technique has the unique ability to reliably exclude such background contributions. The PFY-EXAFS measurement utilizes a large solid-state detector with partial fluorescence energy resolution (≥200 eV) that collects all fluorescent events, from the sample or potentially the surrounding environment. The HERFD-EXAFS measurement employs a large emission spectrometer based on Bragg optics that collects a narrow ∼1 eV bandwidth of the fluorescence emitted from the sample. The spectrometer is aligned and calibrated to the sample's position in space so that only fluorescent events of a selected energy originating from the sample will satisfy Bragg's law at the analyzer crystals and thus be detected. Fluorescence that does not arise from the sample is generally rejected by the spectrometer, eliminating the possibility that stray scattering will contribute to the measured EXAFS. In the case of sMMO,^28^ the HERFD-EXAFS technique eliminated metallic iron EXAFS contamination arising from the cryostat and/or other beamline components.

**Fig. 5:**
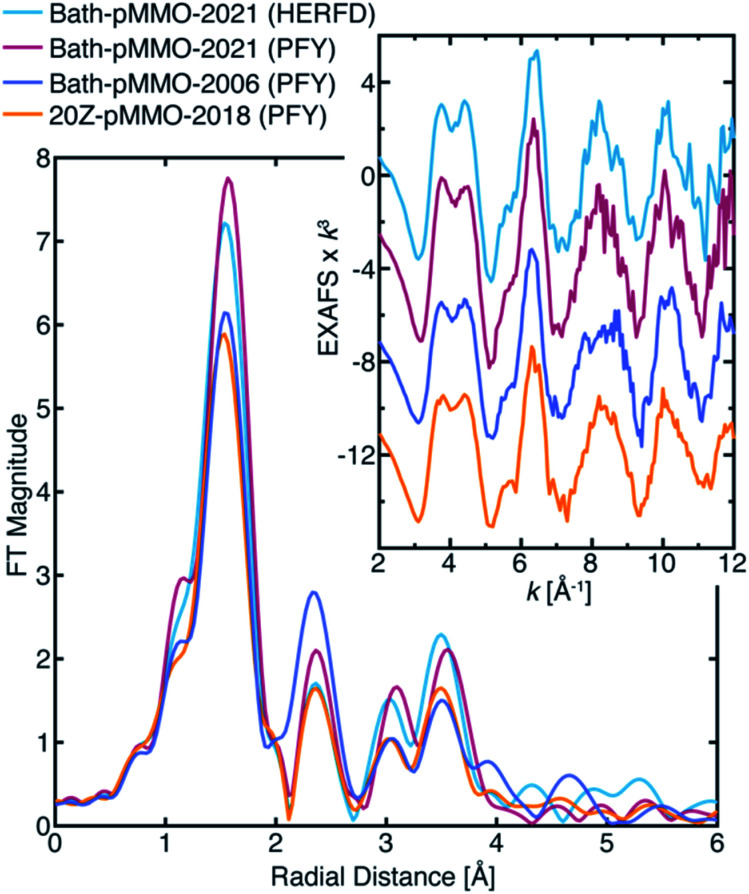
FT-EXAFS of various pMMO samples collected by either the HERFD- or PFY-EXAFS technique. The **Bath-pMMO-2021** samples plotted here are of the high conc. sample set. The FT spectra were calculated from the *k*^3^-weighted EXAFS spectra shown in the inset, over a *k*-range of 2–12 Å^−1^.

The HERFD-EXAFS of **Bath-pMMO-2021** at both high and low protein concentrations are similar and may be considered free of metallic (copper) background contamination, Fig. S6.† Neither spectrum exhibits the previously reported Cu–Cu scattering feature. The HERFD-EXAFS also shows that the protein concentration does not significantly influence the EXAFS signal. Most importantly, the HERFD-EXAFS and the PFY-EXAFS collected here are nearly the same, Fig. 5. Both the HERFD- and PFY-EXAFS of **Bath-pMMO-2021** resemble the EXAFS of **20Z-pMMO-2018** well beyond the first radial shell. The first radial shell of the **Bath-pMMO-2021** HERFD- and PFY-EXAFS appears more intense than that of **20Z-pMMO-2018** (Fig. 5) because the **Bath-pMMO-2021** data were collected with less photodamage, which dampens the first radial shell's intensity (Fig. 4B). In the 2–3 Å region of the FTs, the HERFD-EXAFS spectrum of **Bath-pMMO-2021** is nearly superimposable with the **20Z-pMMO-2018** PFY-EXAFS, Fig. 5. The **Bath-pMMO-2021** (HERFD and PFY) and **20Z-pMMO-2018** FT-EXAFS spectra all exhibit a lower intensity *R* ∼2.3 Å shell compared to the **Bath-pMMO-2006** spectrum.

One potential explanation for the lack of Cu–Cu scattering interaction in the newly collected **Bath-pMMO-2021** PFY-EXAFS is that dinuclear or previously suggested higher nuclearity clusters^9^ may have degraded during isolation and purification. To address this possibility, three additional samples of pMMO were studied by HERFD-EXAFS: whole cell samples of pMMO (**Bath-pMMOcell-2021**), the isolated membrane-bound pMMO protein (**Bath-pMMOmem-2021**), and dithionite-reduced, purified pMMO (**Bath-pMMOred-2021**). None of these samples exhibited a prominent Cu–Cu scattering interaction, as observed previously for **Bath-pMMOred-2006**, Fig. S7.†^16^ The Cu–Cu scattering shell of the **Bath-pMMOred-2006** FT-EXAFS spectrum appeared significant due to the closer-to-equal intensity ratio of the first and second radial shells. The HERFD-EXAFS of **Bath-pMMOred-2021** exhibits a more intense first radial shell in its FT-EXAFS spectrum and a less intense second shell than what was observed for **Bath-pMMOred-2006**. The intensity ratio of the first and second scattering shells is substantially greater for **Bath-pMMOred-2021** than what was observed previously. The low intensity of the first radial shell for the **Bath-pMMOred-2006** could potentially result from photodamage that increases the disorder of the first coordination shell and reduces its amplitude as demonstrated by the photodamage studies of **Bath-pMMO-2021**, Fig. 4. Taken together, these HERFD-EXAFS data indicate that any potential cluster is not lost due to protein purification; a Cu–Cu interaction is not observed in any of these samples.

### Evaluation of a metallic background contribution

The differences between the EXAFS of **Bath-pMMO-2006** and **Bath-pMMO-2021** (PFY- and HERFD-EXAFS), Fig. 5, could indicate that the samples studied here are inherently different. However, this conclusion does not account for variable or spurious background signal contributions. While obtaining parallel HERFD- and PFY-EXAFS spectra on the same sample preparations allowed us to verify the absence of any Cu metallic background in the present data, the possibility of a metallic background contribution in the previously reported data requires further assessment. To investigate if metallic copper scattering could be a contributing factor, two methods were used: (i) collecting PFY-EXAFS under conditions in which potential background contributions to the spectra could be maximized (*e.g.* by allowing the beam spot to move slightly off the ideal position on the sample and by exposing more of the sample, sample holder, and cryostat), and (ii) digitally contaminating the EXAFS spectrum with a metallic copper foil.

**Fig. 6:**
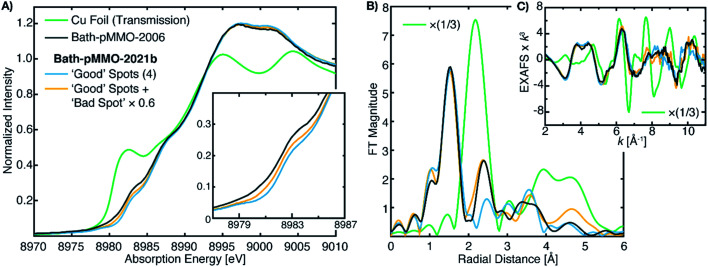
(A) Cu PFY-XAS of **Bath-pMMO-2021b** (high conc.) collected at five different sample spots under “non-ideal conditions,” including full-flux, no slitting of the beam spot (beam spot 8(h) × 2(v) mm), and poor alignment to the sample to partially overshoot sample edges. Averages of the “good” spots with and without the “bad” spot scan (scaled by 0.6) exhibit different intensities at 8983 eV. (B) Non-phase shifted FT-EXAFS spectra of Cu foil, previously published **Bath-pMMO-2006**,^16^ and **pMMO-Bath-2021** “good” spot averages with and without “bad” spot (scaled by 0.6). FT spectra were taken from the (C) raw *k*^3^-weighted EXAFS over a *k* range 2–11 Å^−1^.

Attempts to collect PFY-EXAFS under non-ideal conditions on a single duplicate sample of pMMO (**Bath-pMMO-2021b**) were met with variable results, Fig. 6. The PFY-XAS under full-flux conditions (at saturating photodamage conditions) and a maximum beam spot size yielded scans at four sample spots that appeared “good,” the average of which is again consistent with the photodamaged **Bath-pMMO-2021** observed above (Fig. 4) and the EXAFS of **20Z-pMMO-2018**. However, a single sample spot (Fig. S8†) did produce a more intense shoulder at ∼8983 eV, and inclusion of this scan in the average yields an edge that more closely resembles **Bath-pMMO-2006**, Fig. 6. In order to better visualize the potential Cu metallic background contribution, the transmission XAS spectrum of Cu foil is also shown in Fig. 6. The Cu foil has a prominent feature at approximately the same energy, 8983 eV, as the previously reported **Bath-pMMO-2006** data and the newly reported **Bath-pMMO-2021b** data when the “bad” spot was intentionally included in the average. Thus, these data suggest that a metallic Cu background will broaden the pre-edge feature at 8978 eV and increase the intensity of the observed 8983 eV “photodamage” feature.

The average of the four good spots of **Bath-pMMO-2021b** yields a raw PFY-EXAFS spectrum that resembles that of **20Z-pMMO-2018** and the newly collected **Bath-pMMO-2021** EXAFS. While the FT-EXAFS spectrum of the good spots of **Bath-pMMO-2021b** does not exhibit the Cu–Cu scattering interaction, inclusion of a bad spot into the average of **Bath-pMMO-2021b** significantly perturbs the PFY-EXAFS signal in both the raw and FT spectra, Fig. 6 and S8.† The classic camelback pattern of the histidine imidazole scattering is preserved at *k* ∼ 4 Å^−1^, but at higher *k*, a modulation of the EXAFS pattern is observed that matches the transmission EXAFS spectrum of copper foil. The EXAFS have an acceptable signal/noise ratio until a *k*-value of 11 Å^−1^, setting an upper limit for the calculation of FT spectra.

Although the background contribution is not quantifiable or controllable by this methodology, it is clear that the inclusion of background signals into the averaging of the EXAFS spectrum can have pronounced effects. In an effort to further replicate the FT-EXAFS spectrum of **Bath-pMMO-2006**, the contribution of this bad spot was scaled to 60% of its normalized intensity prior to averaging of the five spots. The resultant EXAFS spectrum yields a second radial shell that matches both the radial distance and the intensity of the Cu–Cu scattering shell of **Bath-pMMO-2006**. It is important to note that the second radial shell position in different averages is not static and begins to match Cu foil with the inclusion of higher background contributions, Fig. S8.† Thus, due to the complexity of the various EXAFS scattering interactions, a simple observation of the radial position of the FT spectrum is not sufficient to exclude the possibility of metallic background contributions.

**Fig. 7:**
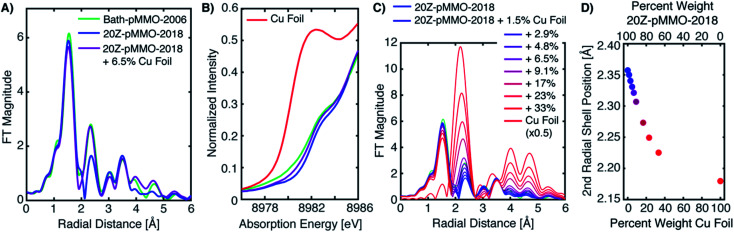
(A) Non-phase shifted FT-EXAFS of **Bath-pMMO-2006** and **20Z-pMMO-2018**. A 0.07 weighted normalized XAS signal of Cu foil signal was added to the normalized XAS signal of **20Z-pMMO-2018** to yield a digitally contaminated spectrum: **20Z-pMMO-2018** + 6.5% Cu Foil. (B) The low-energy region of the Cu K-edge XAS spectra of A are shown along with the pure Cu metal spectrum. (C) Non-phase shifted FT-EXAFS of **20Z-pMMO-2018** with increasing amounts of Cu foil contamination. The final percent weight Cu foil for each spectrum is given in the legend. (D) The radial position of the second shell for the digital contamination series plotted in C. All FT spectra were taken from the *k*^3^-weighted EXAFS over a *k*-range of 2–12 Å^−1^.

Because the above approach did not exactly reproduce the previously published EXAFS and the Cu–Cu scattering feature in question, we also tested the possibility of metallic background scattering through digital contamination of the EXAFS signal. Utilizing the EXAFS of **20Z-pMMO-2018** (processed up to a *k*-value of 12 Å^−1^), the same EXAFS signal was digitally contaminated with the copper foil EXAFS signal. The addition of the Cu foil spectrum to **20Z-pMMO-2018** to yield a summed spectrum that contain ∼6.5% Cu foil by weight (**20Z-pMMO-2018** + 6.5% Cu Foil), is presented in Fig. 7A. The FT-EXAFS spectrum of **20Z-pMMO-2018** + 6.5% Cu Foil is very similar to the previously published spectrum of **Bath-pMMO-2006**. In particular, the Cu–Cu feature of **Bath-pMMO-2006** is well reproduced by the addition of a small percentage of Cu foil. In addition, the normalized XAS spectrum of **20Z-pMMO-2018** + 6.5% Cu Foil and that of **Bath-pMMO-2006** (ref. 16) both exhibit a broadened and poorly resolved pre-edge feature at ∼8979 eV compared to the more well-resolved feature of **20Z-pMMO-2018**, Fig. 7B.^23^ Furthermore, the small percentage of Cu foil subtly increases the intensity of the 8983 eV feature. These broadening and plateauing effects resemble those observed for iron metallic signal contamination in Fe PFY-XAS data collection.^28^

A series of summed EXAFS spectra further elucidates the complex effects of metallic copper on the pMMO EXAFS (Fig. 7C). At lower levels of Cu foil additions, no significant radial distance shifts and intensity differences are observed for the first coordination shell and longer range scattering interactions at *R* = 2.8–3.8 Å (non-phase shifted) attributed to the imidazole scattering paths of coordinating histidines. The position of the second radial shell exhibits clear sensitivity to the amount of the Cu foil present in the summed spectrum, however, Fig. 7C. Plotting the percent weight of Cu foil in the summed spectra *versus* the position of the second radial shell, Fig. 7D, shows that the radial distance may shift as much as ∼0.05 Å with the presence of ∼9% metallic copper in the spectrum. Lastly, new weak features in the FT of **20Z-pMMO-2018** + 6.5% Cu Foil are observed at radial distances greater than 3.8 Å that also match features observed in **Bath-pMMO-2006**, Fig. 7A and C. These longer-range scattering shells thus derive from the metallic Cu background. This summation exercise demonstrates that the simple comparisons of radial FT shell positions of standards (*i.e.* Cu foil) with experimental spectra, as utilized previously,^16^ can be misleading and is an insufficient method for assessing the presence of a potential metallic background signal.

### EXAFS modeling of Bath-pMMO

The newly collected EXAFS data of **Bath-pMMO-2021** do not exhibit the previously observed Cu–Cu scattering interaction that is consistent with copper metallic background scattering in the previously reported data. The similarities between the EXAFS spectra of **20Z-pMMO-2018** and **Bath-pMMO-2021** collected here suggest that these two pMMOs from different methanotrophs have similar average copper coordination environments. Recent EPR and ENDOR studies of Bath-pMMO reveal the presence of an exchangeable water at the Cu_B_ site.^21^ Furthermore, the Cu_C_ site is also suggested to coordinate a water species. This information was not available at the time of the **20Z-pMMO-2018** EXAFS analysis, and the PmoC subunit was disordered in the 20Z-pMMO structure, precluding observation of the Cu_C_ site.^23^ With these new data points and the understanding that the previous Cu–Cu feature of **Bath-pMMO-2006** could be due to metallic scattering, we revisited the EXAFS modeling of both **Bath-pMMO-2021** and **20Z-pMMO-2018**.

**Fig. 8:**
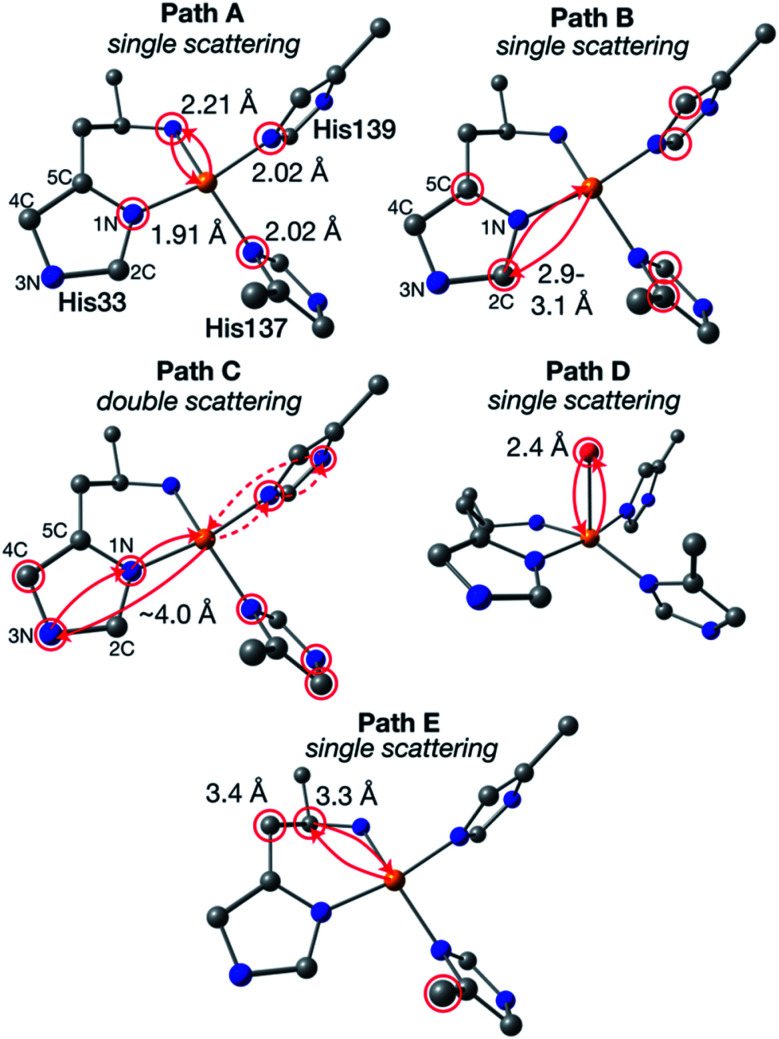
Generalized EXAFS scattering paths determined for the Cu_B_ site. The calculated model is taken from ref. 17. Atom colors: Cu, orange; N, blue; C, gray; O, red; H omitted. Atoms of degenerate scattering paths are circled in red. Examples of both scattering directions of path C are shown in solid and dashed lines.

**Fig. 9:**
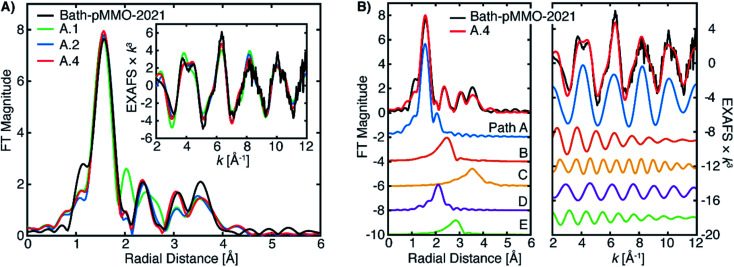
(A) Selected EXAFS fits of **pMMO-Bath-2021** with EXAFS parameters detailed in Table 2. The data were fit of over an *R*-range of 1–4 Å, and the FT-EXAFS was calculated from a *k*-range of 2–12 Å^−1^. (B) The individual fitted EXAFS paths of Fit A.4.

Previous quantum refinement of the Bath-pMMO crystal structure supported a monocopper Cu_B_ site with a possible coordinating water.^17^ We have used this structure as a basis for modeling the pMMO EXAFS and calculation of theoretical EXAFS scattering paths. Numerous paths of effective scattering distances between ∼1.9 and 5.0 Å were found, and can be classified into five major groups, Fig. 8. The first coordination sphere is comprised of the coordinating nitrogenous ligands yielding 4 Cu–N single scattering paths (Fig. 9, path A). The coordinating histidines have an additional single Cu–C single scattering path interaction at a *R*_eff_ distance of 2.9–3.1 Å, for each of the 2C and 5C centers of the imidazole ring (path B). At a longer *R*_eff_ distance (∼4.0–4.2 Å), the multiple scattering paths of the coordinating and remote 3N/4C atoms are found (path C). The forward scattering character of these paths contributes significantly to the EXAFS. We also consider a coordinating water found at *R*_eff_ 2.4 Å despite an anticipated large amount of disorder typical for weakly coordinated water molecules (path D). Finally, the bidendate ligation of His-33 creates additional Cu–C single scattering paths of *R*_eff_ ∼3.3 Å (path E). These five theoretical paths are the basis for the EXAFS modeling and fitting described below. We also consider Cu_C_ as a 2His1Asp coordination site. Its similar histidine environment will change the average coordination number employed, and the aspartic acid will have similar first coordination sphere scattering distances as path A.

The **Bath-pMMO-2021** PFY EXAFS is well modeled and fit using the calculated paths above. The first coordination sphere is represented by a Cu–N/O scattering interaction of four-fold degeneracy at a mean distance of 2.0 Å and a moderately low Debye–Waller (*σ*^2^) value. The coordination number *N* and *σ*^2^-values generally influence the intensity of the FT-EXAFS features with an inverse relationship, and given the difference of intensities observed for the first radial shell of **Bath-pMMO-2021** compared to previous data,^16,23,27^Fig. 5, inherent differences in the fitted parameters are anticipated. The intensity of the **Bath-pMMO-2021** first shell is well modeled with a larger *N* = 4, Table 2, rather than the previously *N* = 2.5 employed for **Bath-pMMO-2006** and **20Z-pMMO-2018**.^16,23,27^ The larger *N* used here better reflects the currently proposed coordination environment of pMMO.^21^ Furthermore, a comparable, if not smaller, *σ*^2^-value for path A of **Bath-pMMO-2021** is obtained compared to previous fits.^16,23,27^ General histidine scattering interactions are well modeled (paths B and C) at both distances and disorder factors typical of such interactions.^36,37^ The inclusion of only the histidine interactions poorly models the observed EXAFS response, Fit A.1, Table 2. In particular, the additional radial feature at *R* ∼2 Å of the fit does not match the experiment, Fig. 9A. The fit is improved significantly by the inclusion of a Cu–O scattering interaction at approximately 2.55 Å, path D, Fit A.2. Paths B and D are out-of-phase with one another at lower *k* values and in-phase at *k*-values greater than 6 Å^−1^, Fig. 9B. The interactions of paths B and D yield the valley of intensity between the first and second radial shell observed in the FT of the experimental data. Most importantly, the inclusion of the shorter Cu–O interaction (path D) and the interaction of these two paths yields the weak second-shell at a radial distance of *R* = 2.35 Å, but does not yield the higher intensity of the Cu–Cu interaction previously observed and modeled for **Bath-pMMO-2006**. Although quantum refinement of the Cu_B_ site found Cu–O(water) distances in the range of 2.20 to 2.34 Å,^17^ the modeled EXAFS interaction of Cu–O may also reflect interactions in the Cu_C_ site that are poorly understood and not structurally characterized. While Fit A.2 utilizes *N* = 4 for the fitting of path B, increasing this to *N* = 6 better reflects the possible degenerate scattering paths of the Cu_B_ site and a comparable fit can be obtained, Fit A.3, showing that the EXAFS is less sensitive to coordination number of this interaction. The **Bath-pMMO-2021** fit is further refined by the inclusion of a final Cu–C interaction, path E, at approximately 3.35 Å, Fit A.4.

**Table 2:** EXAFS fit parameters and statistics for **Bath-pMMO-2021**

Fit	Path^a^	*N*	*R* (Å)	±	*σ* ^2^ (Å^2^)	±	Δ*E*_0_^b^	*χ* c
A.1	Cu–N/O (A)	4	1.995	0.011	0.00367	0.00089	−2.294	93.72
	Cu–C (B)	4	3.014	0.047	0.00921	0.00599		
	Cu–N_his_ (C)	10	4.188	0.038	0.00666	0.00415		
A.2	Cu–N/O (A)	4	1.992	0.008	0.00344	0.00061	−3.615	46.63
	Cu–C (B)	4	2.972	0.030	0.00841	0.00378		
	Cu–N_his_ (C)	10	4.177	0.028	0.00653	0.00285		
	Cu–O (D)	4	2.563	0.023	0.01020	0.00345		
A.3	Cu–N/O (A)	4	1.992	0.008	0.00347	0.00064	−3.652	52.35
	Cu–C (B)	6	2.978	0.034	0.01347	0.00488		
	Cu–N_his_ (C)	10	4.178	0.030	0.00671	0.00310		
	Cu–O (D)	4	2.565	0.022	0.00918	0.00329		
A.4	Cu–N/O (A)	4	2.000	0.008	0.00356	0.00064	−1.808	50.08
	Cu–C (B)	6	3.020	0.035	0.01339	0.00493		
	Cu–N_his_ (C)	10	4.189	0.029	0.00677	0.00310		
	Cu–O (D)	2	2.572	0.021	0.00294	0.00237		
	Cu–C (E)	4	3.383	0.046	0.01029	0.00700		
A.5	Cu–N/O (A)	4	1.999	0.011	0.00266	0.00072	−1.330	62.93
	Cu–C (B)	6	3.020	0.068	0.01644	0.00824		
	Cu–NN (C)	10	4.194	0.035	0.00677	0.00353		
	Cu–O (D)	2	2.551	0.074	0.00771	0.01631		
	Cu–C (E)	4	3.390	0.055	0.01010	0.00795		
	Cu–Cu	0.25	2.569	0.110	0.00437	0.00684		

a The scattering paths in parentheses correspond to those depicted in Fig. 8.

b The fitted Δ*E*_0_ values are shifts in eV relative to the set *E*_0_ value of 8990.0 eV.

c The reduced *χ*^2^ is normalized for the number of variables used in the fit.

The fitted model of A.4 reproduces the experimental data of **Bath-pMMO-2021** well (Fig. 9). Additionally, this model is very similar to that originally employed for the fitting of **Bath-pMMO-2006**,^16^ with the omission of the Cu–Cu interaction. Building off of Fit A.4, the inclusion of a short Cu–Cu scattering interaction with a low coordination number of *N* = 0.25 yields a statistically poorer fit of the data (Fit A.5, Table 2, Fig. S9†) than A.4 as evaluated by the larger *χ*^2^ value for Fit A.5. The *σ*^2^-value for the Cu–Cu scattering path is also fairly large at 6.8 × 10^−3^ Å^2^ and would physically represent a fairly disordered Cu–Cu scattering interaction, if it were present. Thus, the inclusion of a Cu–Cu scattering path in the fitting of **Bath-pMMO-2021** does not appear to be warranted.

**Fig. 10:**
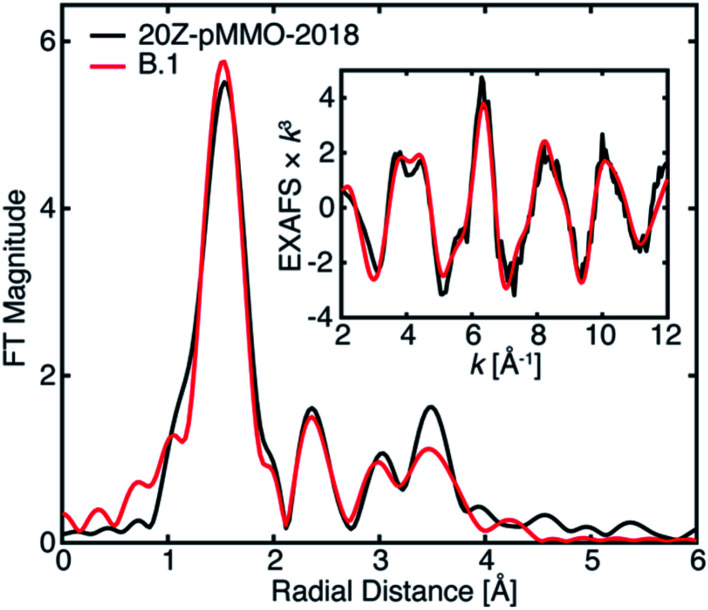
Best EXAFS fitting of **20Z-pMMO-2018** as detailed in Table 3. The data were fit over an *R*-range of 1–4 Å, and the FT-EXAFS was calculated from a *k*-range of 2–12 Å^−1^.

The EXAFS of **20Z-pMMO-2018** generally resembles that of **Bath-pMMO-2021** except for their significant intensity differences of the first radial shells in the FT, Fig. 5. As detailed above, photodamage of **Bath-pMMO-2021** decreases the first radial shell's intensity as a function of dose, eventually yielding an EXAFS spectrum matching that of **20Z-pMMO-2018**, Fig. 4B. The **20Z-pMMO-2018** EXAFS can be fit with a similar model to that of **Bath-pMMO-2021** with the exception of a lower coordination number for path A, Fit B.1, Table 3, Fig. 10. The decreased coordination number yields a similar *σ*^2^-value to that fitted for **Bath-pMMO-2021** (Table 2, Fit A.4). However, it is also possible that the coordination number remains closer to four, and the increase in *σ*^2^-value reflects the increased static disorder due to photodamage-mediated Cu–X bond cleavage, Fit B.2, Table 3.

**Table 3:** EXAFS fit parameters and statistics for **20Z-pMMO-2018**

Fit	Path^a^	*N*	*R* (Å)	±	*σ* ^2^ (Å^2^)	±	Δ*E*_0_^b^	*χ* c
B.1	Cu–N/O (A)	3	1.972	0.008	0.00352	0.00057	−5.374	60.15
	Cu–C (B)	6	2.966	0.025	0.00850	0.00279		
	Cu–N_his_ (C)	10	4.152	0.028	0.00907	0.00291		
	Cu–O (D)	2	2.546	0.028	0.00901	0.00414		
	Cu–C (E)	4	3.364	0.079	0.02497	0.01685		
B.2	Cu–N/O (A)	4	1.971	0.011	0.00573	0.00082	−6.003	105.58
	Cu–C (B)	6	2.985	0.039	0.01446	0.00525		
	Cu–N_his_ (C)	10	4.142	0.037	0.00938	0.00400		
	Cu–O (D)	2	2.533	0.039	0.00951	0.00594		
	Cu–C (E)	4	3.359	0.063	0.01452	0.01045		

a The scattering paths in parentheses correspond to those depicted in Fig. 8.

b The fitted Δ*E*_0_ values are shifts in eV relative to the set *E*_0_ value of 8990.0 eV.

c The reduced *χ*^2^ is normalized for the number of variables used in the fit.

**Fig. 11:**
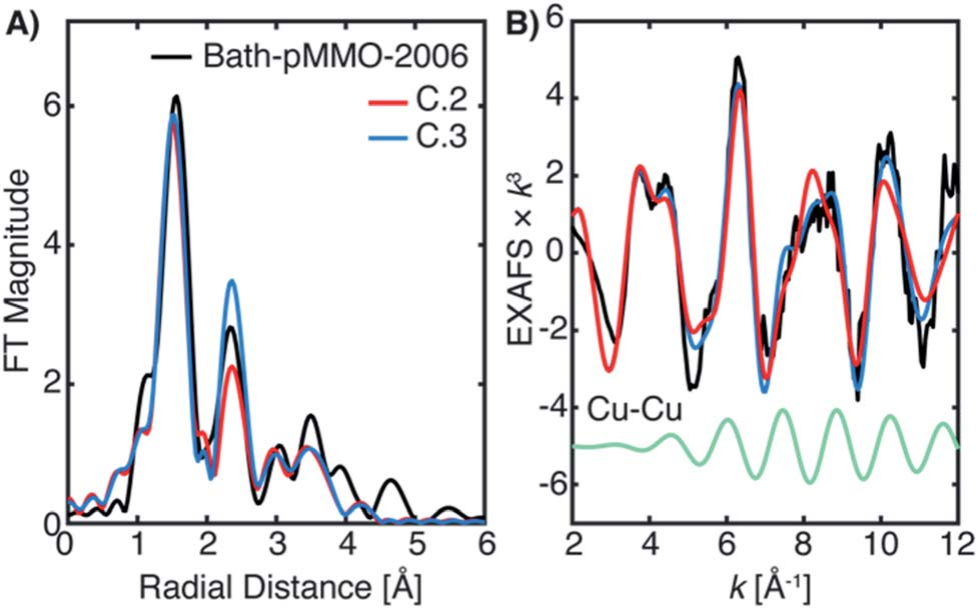
(A) EXAFS fitting of **Bath-pMMO-2006** using the best-fit parameters of **20Z-pMMO-2018** (Fit B.1, Table 3) and with an additional 2.56 Å Cu–Cu scattering interaction (Fit C.3), Table 4. The data were fit over an *R*-range of 1–4 Å, and the FT-EXAFS was calculated from a *k*-range of 2–12 Å^−1^. (B) The *k*^3^-weighted EXAFS of **Bath-pMMO-2006** overlaid with fits C.2 and C.3. The individual Cu–Cu scattering path of Fit C.3 (green) is offset to highlight its contribution. The data were fit over an *R*-range of 1–4 Å, and the FT-EXAFS was calculated from a *k*-range of 2–12 Å^−1^.

**Fig. 12:**
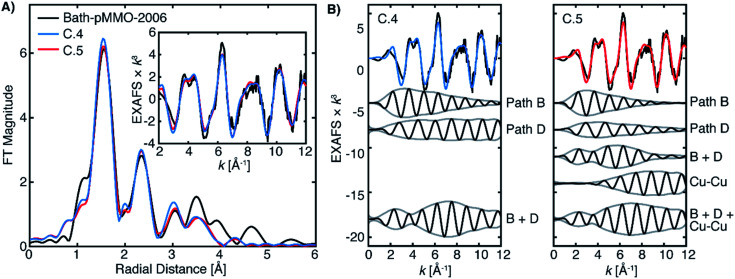
(A) Fits C.4 and C.5 of **Bath-pMMO-2006** as detailed in Table 4. The FT spectra were calculated over a *k*-range of 2–12 Å^−1^. (B) Selected fitted paths of fits C.4 and C.5 and summations of their signal envelopes.

The **20Z-pMMO-2018** fits differ slightly from those reported in Ro *et al.*^23^ as the shorter Cu–O interaction of path D was not considered previously. However, the pMMO model employed here for fitting the EXAFS of **Bath-pMMO-2021** and **20Z-pMMO-2018** is very similar to that used for the fitting of **Bath-pMMO-2006** with the exception of the Cu–Cu scattering interaction, Fit C.1, Table 4. **20Z-pMMO-2018** and **Bath-pMMO-2006** have similar FT-EXAFS spectra except for the radial shell at *R* ∼2.3 Å corresponding to the previously assigned 2.51 Å Cu–Cu scattering interaction in **Bath-pMMO-2006**.^16^ The EXAFS of **Bath-pMMO-2006** was initially fit with the best fit parameters from Fit B.1 for **20Z-pMMO-2018**, restricting the effective *R* and *σ*^2^-values for each path and only allowing Δ*E*_0_ to be fitted. The result (Fit C.2) partially fits the **Bath-pMMO-2006** data in the first-shell and long-range scattering interactions of the FT-EXAFS spectrum (Fig. 12A). The intensity of the radial feature at 2.3 Å is not matched, and the fit has a sharper feature at *k* ∼8 Å^−1^ in the *k*^3^-weighted EXAFS compared to the experimental data (Fig. 11B). When a 2.56 Å Cu–Cu scattering interaction calculated from metallic copper with a partial coordination number of *N* = 0.5 is added to the constrained fit of C.2, the resultant fit, C.3, has a Cu–Cu refined distance of 2.56 Å with a *σ*^2^-value of 5.3 × 10^−3^ Å^2^, and the FT-EXAFS exhibits increased intensity of the 2.3 Å radial shell, Fig. 11A. For metallic copper, the degeneracy of this calculated path is *N* = 12; the reduced *N* = 0.5 employed in the fit represents a fraction (∼4%) of Cu metallic contribution. Furthermore, the Cu–Cu scattering interaction deconstructively interacts with and broadens the pMMO EXAFS signal at *k* ∼8 Å^−1^, Fig. 11B.

**Table 4:** EXAFS fit parameters and statistics for **Bath-pMMO-2006**

Fit	Path^a^	*N*	*R* (Å)	±	*σ* ^2^ (Å^2^)	±	Δ*E*_0_^b^	*χ* c
C.1^d^	Cu–N/O	2.5	1.97		0.00400			
Original	Cu–O/N	0.5	2.22		0.00453			
	**Cu–Cu**	**0.25**	**2.51**		**0.00465**			
	Cu–C	1.0	3.36		0.00339			
	Cu–C	2.5	3.95		0.00456			
C.2	Cu–N/O (A)^e^	3	1.972		0.00352		−6.444	1.41
	Cu–C (B)^e^	6	2.966		0.00850			
	Cu–N_his_ (C)^e^	10	4.152		0.00907			
	Cu–O (D)^e^	2	2.546		0.00901			
	Cu–C (E)^e^	4	3.364		0.02497			
C.3	Cu–N/O (A)^e^	3	1.972		0.00352		−6.028	0.94
	Cu–C (B)^e^	6	2.966		0.00850			
	Cu–N_his_ (C)^e^	10	4.152		0.00907			
	Cu–O (D)^e^	2	2.546		0.00901			
	Cu–C (E)^e^	4	3.364		0.02497			
	Cu–Cu	0.5	2.564	0.016	0.00530	0.00163		
C.4	Cu–N/O (A)	3	1.982	0.008	0.00310	0.00067	−4.597	1.11
	Cu–C (B)	6	2.989	0.026	0.01018	0.00297		
	Cu–N_his_ (C)	10	4.179	0.042	0.01136	0.00494		
	Cu–O (D)	2	2.581	0.012	0.00033	0.00126		
	Cu–C (E)	4	3.342	0.051	0.01358	0.00862		
C.5	Cu–N/O (A)	3	1.982	0.012	0.00307	0.00072	−4.306	1.25
	Cu–C (B)	6	2.980	0.068	0.01731	0.00759		
	Cu–N_his_ (C)	10	4.179	0.047	0.01109	0.00512		
	Cu–O (D)	2	2.517	0.054	0.00782	0.01345		
	Cu–C (E)	4	3.368	0.065	0.01430	0.01067		
	Cu–Cu	0.5	2.572	0.030	0.00312	0.00212		

a The scattering paths in parentheses correspond to those depicted in Fig. 8.

b The fitted Δ*E*_0_ values are shifts in eV relative to the set *E*_0_ value of 8990.0 eV.

c The reduced *χ*^2^ is normalized for the number of variables used in the fit.

d Data taken from Fit 6.5 in Lieberman, *et al.*^16^ No error estimates were reported.

e The fitted *R* and *σ*^2^ values for these paths are from Fit B.1. The Δ*E*_0_ was allowed to be refined along with the Cu–Cu scattering path's *R* and *σ*^2^-value. There is no calculated error for the set path.

It is evident from the differences between these fits (C.2 and C.3) for **Bath-pMMO-2006** that fitting of the radial feature at *R* ∼2.3 Å is sensitive to the contributions at higher *k*-space from an interaction like Cu–Cu scattering (Fig. 11B). Neither **Bath-pMMO-2021** nor **20Z-pMMO-2018** completely lacks the radial shell at *R* ∼2.3 Å, but each simply exhibits a weaker contribution than **Bath-pMMO-2006**, indicating that a Cu–Cu scattering interaction was indeed required to best model **Bath-pMMO-2006**. To further understand the Cu–Cu interaction observed in **Bath-pMMO-2006**, the previously locked paths (paths A–E) of fits C.2 and C.3 were allowed to refine their individual distances and *σ*^2^-values, resulting in fits C.4 and C.5, respectively. Fit C.4 employs the same model that was used to fit **Bath-pMMO-2021** and **20Z-pMMO-2018**, while Fit C.5 adds an additional Cu–Cu scattering interaction. Inspection of the individual paths reveals that paths B and D in Fit C.4 are out of phase with one another at low *k* and in phase at higher *k*, Fig. 12. The envelope of the sum of paths B and D shows that the maximum scattering amplitude is not reached until approximately 7 Å^−1^, a characteristic typically associated with heavier 3d transition metal scatterers (*i.e.* Cu–Cu).^38,39^ However, the summation of these light-atom scattering paths demonstrates that it is possible to achieve a weak scattering interaction that resembles a Cu–Cu scattering interaction at an *R* ∼2.3 Å radial distance. The unique interaction of paths B and D explains the weaker intensity of the second radial shell observed and fitted for **Bath-pMMO-2021** and **20Z-pMMO-2018** as compared to **Bath-pMMO-2006**. While **Bath-pMMO-2006** Fit C.4 is similar to Fit A.4 for **Bath-pMMO-2021**, the *σ*^2^-value for path D is extremely small (0.3 × 10^−3^ Å^2^), an order of magnitude smaller than what is anticipated for a well-ordered scattering interaction, *σ*^2^ = 3.0 × 10^−3^ Å^2^ (*N* = 1). This underestimated *σ*^2^-value yields an EXAFS signal that erroneously retains its intensity at higher *k*-values. Therefore, the small *σ*^2^-value of path D of Fit C.4 is overcompensating for intensity of the *R* ∼2.3 Å feature, and a Cu–Cu interaction must be included (Fit C.5, Table 4) to obtain a more chemically and physically plausible model.

The fitted Cu–Cu scattering path of C.5 begins to gain intensity at approximately 6 Å^−1^ and remains level, Fig. 12C. The sum of paths B, D and the Cu–Cu interaction in Fit C.5 yields similar EXAFS interactions to the summation of paths B and D for Fit C.4, both exhibiting an identical signal envelope, Fig. 12. These three paths of Fit C.5 (paths B, D and the Cu–Cu) produce the radial shell at 2.3 Å previously assigned only to the Cu–Cu interaction in **Bath-pMMO-2006**. The signal envelopes of the selected path summations for fits C.4 and C.5 are nearly identical, Fig. 12. This analysis demonstrates that the phase relationship of paths B and D produces a unique EXAFS signal that may be inadvertently assigned to a weak Cu–Cu interaction through Fourier transforms or wavelet analysis methods.^40,41^

The fitted distance of the Cu–Cu interaction does not deviate significantly from the calculated *R*_eff_ mean scattering distance for metallic copper, consistent with the hypothesis that the Cu–Cu interaction in this model is from a background metallic contribution rather from the sample itself. In addition, the C.4 and C.5 fits of **Bath-pMMO-2006** are quite similar to the EXAFS fit of the digitally contaminated sample, **20Z-pMMO-2018** + 6.5% Cu foil (Fits D.1 and D2, Table S1†).

In summary, the interaction patterns of paths B and D allow for the successful modeling and reproduction of the weak radial feature at *R* ∼2.3 Å observed in **Bath-pMMO-2021** and **20Z-pMMO-2018**. The best fits for these samples do not include a Cu–Cu interaction and when attempts were made to include a short Cu–Cu interaction for **Bath-pMMO-2021**, no improvement was observed (Fit A.5, Table 2). However, the *R* ∼2.3 Å feature in **Bath-pMMO-2006** cannot be reproduced satisfactorily from the paths B and D alone; **Bath-pMMO-2006** requires the inclusion of a short Cu–Cu scattering interaction to appropriately account for the intensity of the radial feature. The XAS edge analysis, inspection of the FT-EXAFS (Fig. 7) of the digitally contaminated **20Z-pMMO-2018** compared with **Bath-pMMO-2006**, paired with this complete EXAFS model supported by previous computational^17^ and spectroscopic studies^21^ strongly suggests that background copper scattering contributed to the Cu–Cu radial feature of **Bath-pMMO-2006**.

## Conclusions

XAS analysis of the Cu K-edge is an excellent tool to assess copper oxidation state, but Cu(ii) is easily photoreduced to Cu(i) under the damaging conditions of synchrotron radiation, necessitating special precautions to collect undamaged or minimally damaged spectra. The XAS of **Bath-pMMO-2021** was collected under very low flux conditions and relatively fast monochromator scans rates to minimize photodamage, yielding an undamaged Cu K-edge XAS spectrum for Bath-pMMO. This damage-free spectrum exhibits predominately Cu(ii) character with no appearance of an 8983 eV feature previously assigned to Cu(i) in various pMMO samples.^15,16,18,23,27^ These observations are consistent with the EPR quantifications. Subsequent and intentional photodamage of Bath-pMMO to mimic previously reported data shows the growth of a feature at 8983 eV, indicating that this Cu(i) species may not be representative of the protein's native Cu(i) state. Taken together, these observations reconcile apparent discrepancies between copper oxidation state distribution and XAS spectra in previous studies.

HERFD- and PFY-EXAFS of Bath-pMMO do not support a dinuclear copper site. The present EXAFS data are in excellent agreement with those of **20Z-pMMO-2018**,^23^ but contradict the previously observed EXAFS of all other pMMO samples.^16^ While traditional PFY-EXAFS studies of dilute samples may be prone to background contamination signals, the HERFD-EXAFS is a valuable control experiment in which potential metallic copper background contributions are eliminated or extremely suppressed.^28^ The HERFD- and PFY-EXAFS collected here are identical and lack the previously observed Cu–Cu scattering interaction, indicating that the current PFY-EXAFS is also free of background metallic copper contamination. Through both digital contamination of the PFY-EXAFS and collection under non-ideal conditions, we have demonstrated that a metallic copper EXAFS signal may be introduced as a background contribution. This background contribution yields a Cu–Cu radial scattering feature in the FT-EXAFS spectrum similarly to that observed previously. Our analysis shows that simple inspection of the FT-EXAFS is not sufficient to identify a background contribution, but systematic analysis and modeling are required to identify such contributions. Furthermore, we have demonstrated that both photodamage mechanisms and metallic copper contributions influence the Cu K-edge spectrum of pMMO, making deconvolution of these contributions challenging and highlighting the necessity of obtaining photodamage-free XAS data to simplify data analysis. Taken together, this analysis of new and previously reported EPR and XAS data on pMMO provides a more holistic understanding of the apparent discrepancies in previous literature reports. Moreover, the methodological approach presented here establishes clear protocols for more rigorous and quantitative interpretations of XAS data, particularly on dilute metalloproteins. Finally, the work presented herein brings the next major challenge in pMMO research into focus. How a single copper site can form a powerful enough oxidant to directly abstract a proton from methane remains an open question. With the longstanding issue of its active site composition further clarified, the community now has the structural information needed to pursue mechanistic understanding.

## Experimental

### Methanotroph growth and pMMO sample preparation

*M. capsulatus* (Bath) cells were grown in 12 L fermenter batches, and pMMO was isolated, solubilized, and purified by size exclusion chromatography as previously described^21^ (purified into a final buffer of 25 mM PIPES, 250 mM NaCl, 0.02% DDM, pH 7.2). Purified pMMO protein concentrations were determined *via* the colorimetric Bio-Rad DC assay by comparison to BSA standards. pMMO copper-binding stoichiometries were determined *via* inductively coupled plasma-optical emission spectrometry (ICP-OES) at the Quantitative Bio-element Imaging Center at Northwestern University.

Prior to advanced spectroscopic characterization, all pMMO samples were assessed for ^13^C–methane oxidation activity *via* a slightly modified version of the established bicelle activity assay.^23^ Briefly, purified Bath-pMMO samples on ice were diluted to 200 μL of 100 μM protein (protomer concentration). To these samples 50 μL of 30% (w/v) DMPC:CHAPSO 2.8:1 bicelle solution (Molecular Dimensions) were added, and the solution was vigorously pipetted to obtain a homogeneous solution. The samples were then incubated on ice for 30 min, and subsequently diluted with 250 μL of 25 mM PIPES, 250 mM NaCl, pH 7.2, 0.02% DDM. 96 μL of the bicelle-incorporated Bath-pMMO samples were aliquoted into 2 mL screw top reaction vials with septa tops (Agilent). For all reaction samples except “no reductant” negative controls, 4 μL of 7 mM NADH, 25 mM PIPES, 250 mM NaCl, pH 7.2, 0.02% DDM were added; for “no reductant” negative control samples, 4 μL of 25 mM PIPES, 250 mM NaCl, pH 7.2, 0.02% DDM were added instead. A mixture of 2 mL of ^13^C–methane mixed with 1 mL of air was then injected into each septum except for “no methane” negative control samples. All samples were then reacted for 5 min in a water bath held at 30 °C, shaking at 200 rpm. After reaction, the samples were cooled on ice for 5 min, the excess pressure was released, and 500 μL of chloroform spiked with 1 mM dichloromethane was injected into each reaction vial. The reaction vials were shaken at room temperature for 10 min at 2000 rpm and were subsequently centrifuged for 30 min at 2000×*g*, 4 °C. The amount of ^13^C–methanol formed was assessed by gas chromatography-mass spectrometry (GC-MS) monitoring for the 33 *m*/*z*^13^C–methanol fragment ion peak relative to the 49 *m*/*z* dichloromethane internal standard fragment ion peak on an instrumental setup described elsewhere.^23^ Bicelle-incorporated Bath-pMMO specific activities ranged from 9.9 to 12 nmol_methanol_ min^−1^ mg_pMMO_^−1^.

For preparation of purified pMMO EPR and XAS samples, purified pMMO was buffer exchanged *via* PD-10 desalting columns into 25 mM PIPES, 250 mM NaCl, 0.02% DDM, 50% metal-free glycerol, pH 7.2. The buffer-exchanged, purified pMMO was then concentrated by centrifugation (5000×*g*, 4 °C) in a 100 kDa cutoff Amicon centrifugal filter. The concentrated pMMO was then aliquoted into custom quartz Q-band EPR tubes and Kapton wrapped Delrin XAS sample cells and flash frozen in liquid nitrogen, where the samples were stored until the measurements were performed.

For preparation of membrane-bound pMMO XAS samples, isolated *M. capsulatus* (Bath) membranes (533 μM protein concentration) in 25 mM PIPES, 250 mM NaCl, pH 7.2 were aliquoted into XAS sample cells and flash frozen in liquid nitrogen. For preparation of whole cell XAS samples, 1.6 g of frozen *M. capsulatus* (Bath) cells were thawed and washed in 50 mL of 12.2 mM dibasic sodium phosphate, 7.8 mM monobasic sodium phosphate, 5 mM magnesium chloride, pH 7.0 and centrifuged at 4 °C, 10 000×*g*, for 10 min. The supernatant was then removed, and the washing procedure was repeated. The supernatant was again removed, and the cells were resuspended in ∼400 μL of the aforementioned phosphate buffer. The cell suspension was then added directly into the XAS sample cells and flash frozen in liquid nitrogen.

For preparation of the dithionite-reduced purified pMMO XAS samples, 400 μL of 660 μM purified pMMO was deoxygenated *via* Schlenk line and brought into an anaerobic Coy chamber where it was maintained at ∼6 °C. To this pMMO solution, 10 μL of anaerobic 1.312 M sodium dithionite in 25 mM PIPES, 250 mM NaCl, 0.02% DDM, pH 7.2 was added (final concentration of 32 mM dithionite, corresponding to ∼20 : 1 dithionite to Cu). The sample was then incubated for 15 min at room temperature and subsequently aliquoted into XAS sample cells and flash frozen in liquid nitrogen in the anaerobic chamber.

### EPR spectroscopy

Continuous wave (CW) EPR experiments were performed on an X-band Bruker ESP-300 spectrometer featuring an Oxford Instruments ESR-900 cryostat. All spectra were background-corrected by subtraction of a spectrum of Cu(ii)-free buffer (50 mM Tris, 150 mM NaCl, pH 8.0) measured under identical conditions. Cu(ii) spin quantitation was performed by comparison of spectrum double-integral areas to that of a 1 mM CuEDTA standard in 25 mM PIPES, 250 mM NaCl, pH 7.0 measured under identical conditions.

### HERFD XAS measurements

HERFD-XAS measurements were performed at beam line 6–2 at SSRL (3.0 GeV, 500 mA) at 10 K in a liquid helium cryostat. Cu Kα HERFD-XAS were collected using a Si(311) double crystal monochromator upstream for energy selection and a 1 m radius Johann spectrometer for the measurement of X-ray emission equipped with seven Si(444) analyzer crystals and a silicon-drift detector windowed to the Cu Kα emission region. The incident energy was calibrated to the first inflection point of a Cu reference foil set to an energy of 8980.3 eV. Because no Cu reference foil was measured simultaneously during data acquisition, individual spectra were calibrated to a sharp glitch of the Si(311) monochromator occurring at 9235.73 eV, Fig. S10.† A beam size of approximately 400 (h) × 100 (v) μm was used. Cu Kα HERFD-XAS was collected by detection of emitted photons at the maximum of the Cu Kα emission spectrum (∼8048 eV), while scanning the incident energy through the Cu K-edge. A fresh sample spot was utilized for each XAS scan. For the acquisition of undamaged spectra, the sample exposure time was limited and the incident flux was attenuated by the insertion of Al foils into in the beam path.

The raw XAS spectra were initially averaged in Matlab and exported for further processing within Athena.^42^ A second order polynomial was fit to the pre-edge region and subtracted throughout the entire EXAFS spectrum. A three-region cubic spline (with the AUTOBK function) was employed to model the background function to a minimum of *k* = 13 Å^−1^ for all spectra. Fourier transforms were performed over a windowed *k* range detailed in the figure captions, and all FT spectra are presented without a phase shift correction.

### PFY XAS measurements

The final processed *μ*(*E*) XAS data from Lieberman *et al.*,^16^ and Ro *et al.*^23^ were reused here. Their experimental collection procedure is described in detail elsewhere.^16,23^ All EXAFS data could then be processed and presented in a consistent manner (*i.e.* background fitting, windowing, FT parameters, *etc*). Cu K-edge XAS data were recorded on SSRL (3.0 GeV, 500 mA) beamline 9–3 using a 100-element solid state Ge detector (Canberra) as previously described.^43^ The incoming X-rays were selected using a Si(220) double-crystal monochromator and a Rh-coated mirror was utilized for harmonic rejection. A beam spot size of 1.0 mm (v) × 3.0 mm (h) was used throughout, except where noted. Samples were maintained at ∼10 K in a liquid helium flow cryostat. Data were calibrated by simultaneously measuring a copper foil, with the first inflection point set to 8980.3 eV.

Individual PFY-EXAFS scans were evaluated and processed in Matlab to average selected channels of the multi-element detector and to normalize the averaged PFY signal to the incident beam intensity (*I*_0_). These averaged PFY-EXAFS scans were evaluated and processed in Athena for final averaging of multiple scans at different sample positions. All final processed PFY-EXAFS were treated in the same manner as the HERFD-EXAFS detailed above.

### EXAFS modeling

Theoretical EXAFS spectra were calculated using FEFF6 code interfaced through Larch (v. 0.9.47).^44,45^ The EXAFS amplitude, *χ*(*k*), is given by
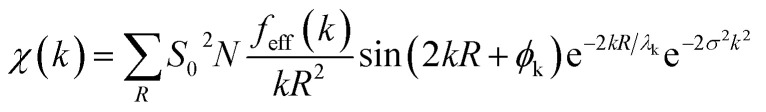
where *S*_0_^2^ is the overall many-body amplitude factor, *N*, is the degeneracy of the path(s), |*f*_eff_(*k*)| is the effective scattering amplitude, and *R* is the absorber-scatterer distance. A Debye-Waller like factor, exp(−2*σ*^2^*k*^2^), is also included to account for disorder. Lastly, *λ*_k_ is the mean free path of the photoelectron and *ϕ*_k_ is the total photoelectron wave phase shift for the interaction between the absorber and the scatterer.

Individual scattering paths were calculated using FEFF6 (ref. 44) and fit to the FT of the EXAFS spectra using Larch.^45^ The FT spectrum of each sample was fit over a range of *R* = 1.0 to 4.0 Å (non-phase shift corrected). The FT is the product of a transform of *k*^3^-weighted EXAFS spectrum with a Hann window over the given *k*-range described in each figure caption. By grouping similar scattering paths of a common coordination shell and summing their degeneracies, *N*, the number of variables used for that coordination shell is minimized to two variables: *σ*^2^ and Δ*R*. A single *E*_0_ variable was used for all paths in a given fit. *S*_0_^2^ was set to 0.9 for all paths. Goodness of final fits were evaluated based on their reduced *χ*^2^ values as calculated within Larch.

## Author contributions

GEC, SD and ACR conceptualized the experiment and all authors participated in the design of the methodology. MOR prepared all samples and performed the EPR and ICP-OES experiments and analysis. GEC collected the XAS data and performed the XAS analysis. GEC and SD developed the XAS analysis approach. GEC wrote the original draft and all authors participated in the review and editing process.

## Conflicts of interest

There are no conflicts to declare.

## Supplementary Material

SC-012-D1SC00676B-s001
