# Supplementary material for: Towards a unified understanding of the copper sites in particulate methane monooxygenase: an X-ray absorption spectroscopic investigation
Source: Chem Sci. 2021 Mar 30;12(17):6194–209. doi: 10.1039/d1sc00676b (PMC8098663; doi:10.1039/d1sc00676b)
Supplement: SC-012-D1SC00676B-s001 [file SC-012-D1SC00676B-s001.pdf]

Electronic Supplementary Material (ESI) for Chemical Science.
This journal is © The Royal Society of Chemistry 2021

Electronic Supplemental Information

for

Towards a unified understanding of the copper sites in particulate methane monooxygenase: An X-ray absorption spectroscopic investigation

George E. Cutsail III,[a,b*] Matthew O. Ross,[c,d] Amy C. Rosenzweig,[c] Serena DeBeer[a*]

a) Max Planck Institute for Chemical Energy Conversion, Stiftstrasse 34-36, D-45470 Mülheim an der Ruhr, Germany

b) University of Duisburg-Essen, Universitätsstrasse 7, D-45151 Essen, Germany

c) Departments of Molecular Biosciences and Chemistry, Northwestern University, Evanston 60208 IL, United States

d) Present address: Department of Chemistry, University of Chicago, Chicago, IL 60637, United States

*Correspondence to: george.cutsail@cec.mpg.de, serena.debeer@cec.mpg.de

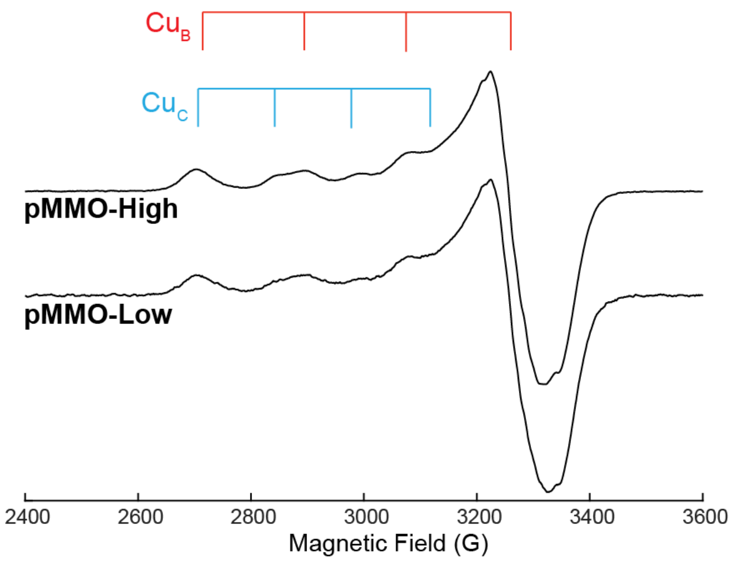

Figure S1. CW X-band EPR spectra of Bath-pMMO XAS samples **Bath-pMMO-2021 (high conc.)** and **Bath-pMMO-2021 (low conc)**. Brackets denote the Cu_B and Cu_C $g_{||}$ and $A_{||}$ values measured previously[1] (Cu_B $g_{||}$ = 2.242, $A_{||}$ = 182 G; Cu_C $g_{||}$ = 2.30, $A_{||}$ = 137 G). Heights normalized to unity for ease of comparison. Collection conditions were as follows: 9.373-9.375 GHz microwave frequency, 320 ms time constant, 12.5 G modulation amplitude, 5 scans, temperature 20 K.

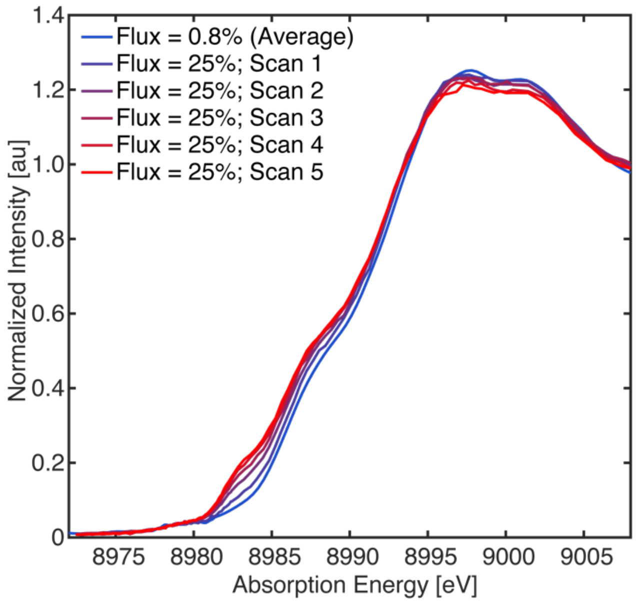

Figure S2. Normalized consecutive damage “short” XAS damage scan of the Cu K-edge of **Bath-pMMO-2021** (low conc.). The low-dose (0.8% flux) scan is the averaged EXAFS of several scans at fresh sample spots as detailed in the experimental section. The consecutive damage scans at 25% available flux were then performed on the same sample at a faster scan speed over the energy range of 8972 to 9010 eV with a total exposure time of 104 s for each scan.

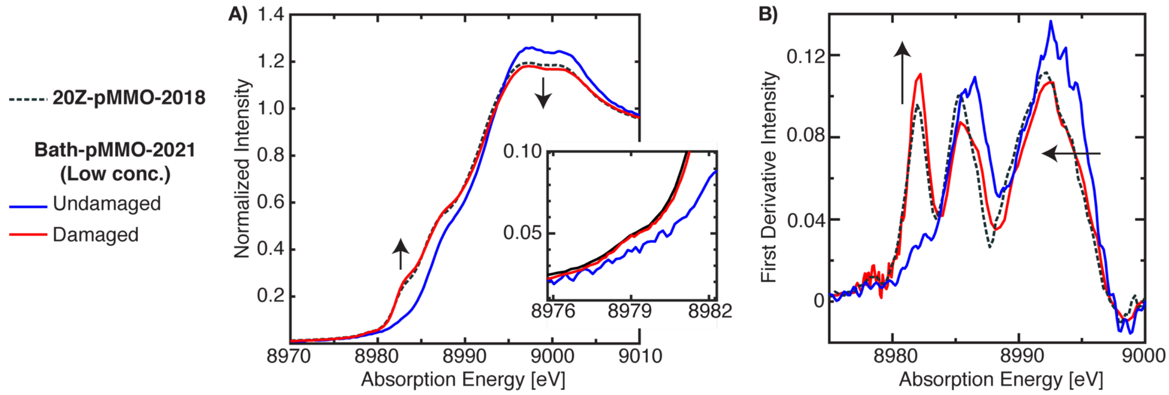

Figure S3. (**A**) Normalized PFY-XAS spectra of **Bath-pMMO-2021** acquired at various photodamage levels. The "undamaged" spectrum is an average of several fresh sample spots at low-dose (0.8%) to achieve adequate signal-to-noise for EXAFS analysis. The "damaged" scan is the full-flux scan after multiple previous passes (the 100% scan as described in Figure 4). Arrows indicate observed trends as a function of photodamage. The data are presented with the normalized XAS spectrum of **20Z-pMMO-2018.**[2] The inset of **A** is an expansion of the pre-edge region. (**B**) The first derivative of the low-energy region of the normalized spectra of panel A.

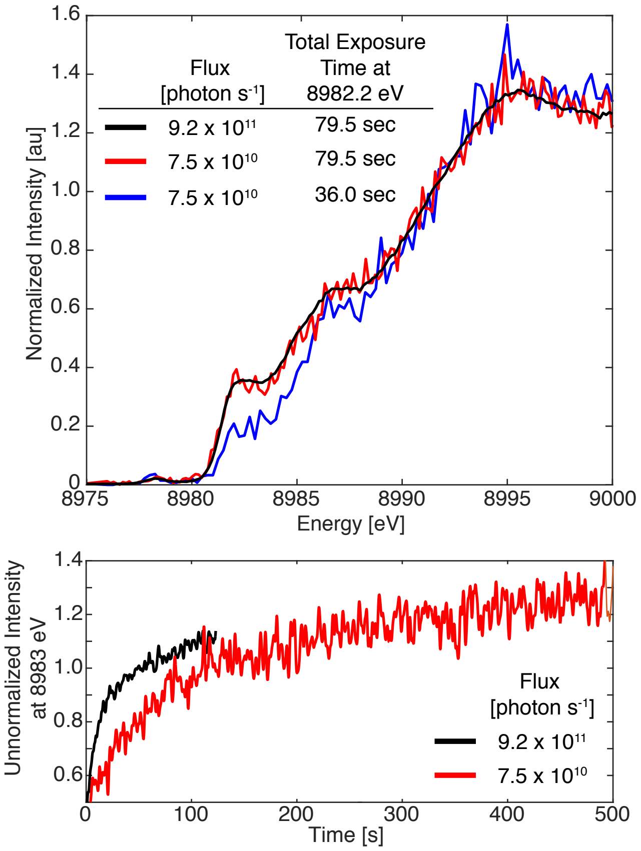

Figure S4. (Top) Cu Kα-HERFD XAS of **Bath-pMMO-2021** collected either at different fluxes or scan rates. (Bottom) Time scans monitoring the Kα fluorescence intensity at a fixed incident energy of 8983 eV. The 8983 eV feature immediately grows in as the photodamage to the sample occurs instantly, but appears to plateau at longer times. The rate of photodamage is retarded by attenuating the flux of incident beam.

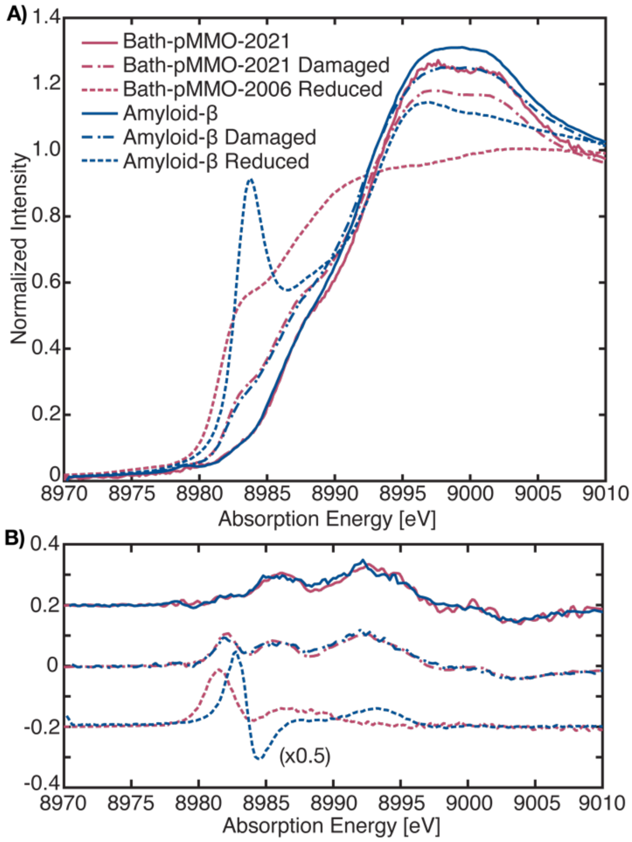

Figure S5. **(A)** PFY Cu-XAS spectra of Bath-pMMO and amyloid-β(1-42) and (**B**) the first derivatives. The undamaged, maximally-damaged, and chemically-reduced spectra of both proteins are shown. A three-point smoothing was applied to the first derivative spectrum of undamaged **Bath-pMMO-2021**; all other derivatives are without smoothing. The Cu XAS data of amyloid-β are taken from Summers et al.[3] The dithionite-reduced spectrum of **Bath-pMMO-2006** is taken from ref.[4]

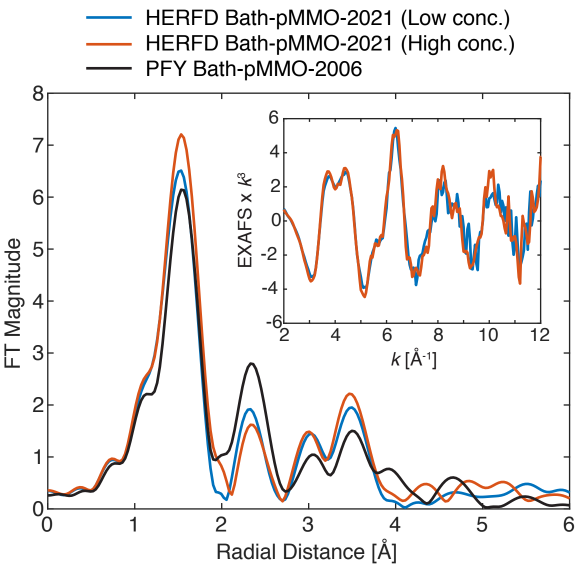

Figure S6. Cu Kα HERFD-EXAFS of **Bath-pMMO-2021**, high and low conc., collected with 4.4 x 10^{11} and 1.2 x 10^{12} photons s^{-1} m^{-2}, respectively. The data is overlaid with the PFY-EXAFS of **Bath-pMMO-2006**[4]. The non-phase shifted FT is taken over a *k*-range of 2 to 12 $Å^{-1}$. The raw unfiltered k^3-weighted HERFD-EXAFS is presented in the figure inset.

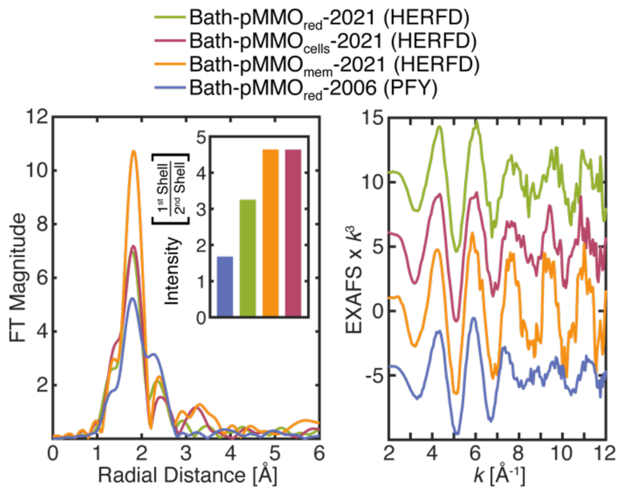

Figure S7. FT-EXAFS (right) and corresponding k^3-weighted EXAFS of **Bath-pMMO$_{red}$-2021**, **Bath-pMMO$_{cell}$-2021**, **Bath-pMMO$_{mem}$-2021** (all HERFD-EXAFS), and **Bath-pMMO$_{red}$-2006** from Lieberman et al (PFY-EXAFS).[4] The inset bar graph displays the ratios of the first (~1.8 Å) and second shell (2.3-4 Å) FT magnitudes. The FT spectra were calculated over a *k*-range of 2-12 $Å^{-1}$. **Bath-pMMO$_{mem}$-2021** exhibits a significantly more intense first radial shell, which may reflect a very well-ordered first-coordination sphere through a substantial decrease in the static disorder of the contributing scattering interactions. Removal of pMMO from the membrane likely destabilizes the copper centers, consistent with the loss of activity upon purification and its recovery using membrane mimetics, such as bicelles or nanodiscs.[5,2] The **Bath-pMMO$_{cell}$-2021** first radial shell exhibits lower intensity, which could be due to many factors, including the presence of other copper proteins and a different copper oxidation state distribution of the pMMO copper centers.

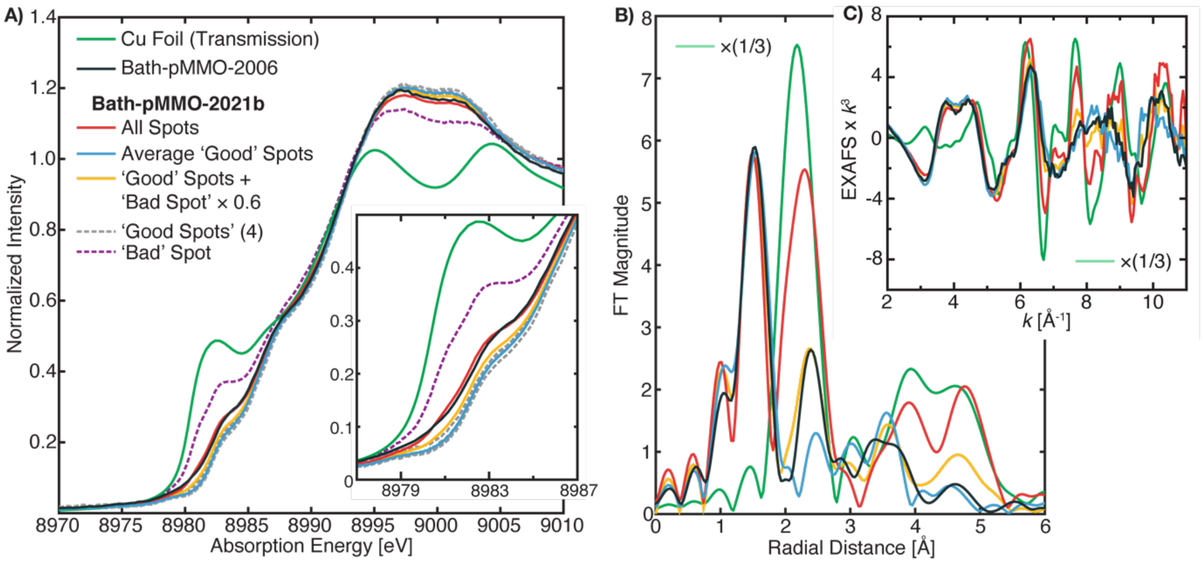

Figure S8. (**A**) Cu PFY-XAS of **Bath-pMMO-2021b** (high conc.) collected at five different sample spots under "non-ideal conditions," including full-flux, no slitting of the beam spot (beam spot 8(h) x 2(v) mm), and poor alignment to the sample to partially overshoot sample edges. The individual 'good' sample spots are plotted in grey dashed lines. The single 'bad' spot plotted in a purple dashed line exhibits an intense feature 8983 eV. Averages of the four 'good' sample spots with and without the 'bad' spot scan (scaled by 0.6) exhibit different intensities at 8983 eV. (**B**) Non-phase shifted FT EXAFS spectra of Cu foil, previously published Bath-pMMO-2006[4], and pMMO-Bath-2021 spot averages with and without the single bad spot. When the relative contribution of the single 'bad' spot is arbitrarily scaled by 0.6 (normalized edge jump), the intensity and width of the radial feature at 2.3 Å matches that of **Bath-pMMO-2006**. The FT spectra were taken from the (**C**) raw k^3-weighted EXAFS over a k range 2-11 Å^{-1}.

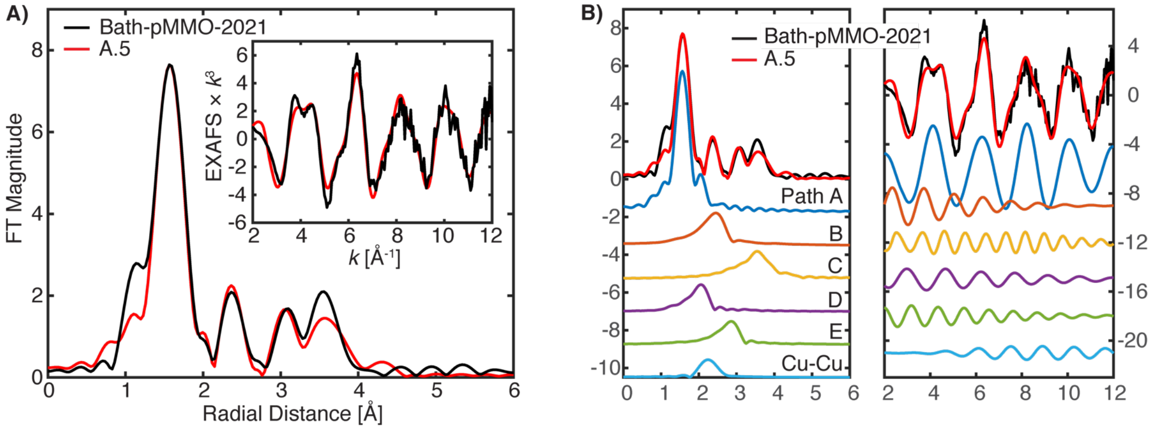

Figure S9. (A) EXAFS fit A.5 of **pMMO-Bath-2021** with EXAFS parameters detailed in Table 2. The data were fit over an *R*-range of 1-4 Å and the FT-EXAFS was calculated from a *k*-range of 2-12 $Å^{-1}$. (B) The individual fitted EXAFS path of fit A.5 are displayed.

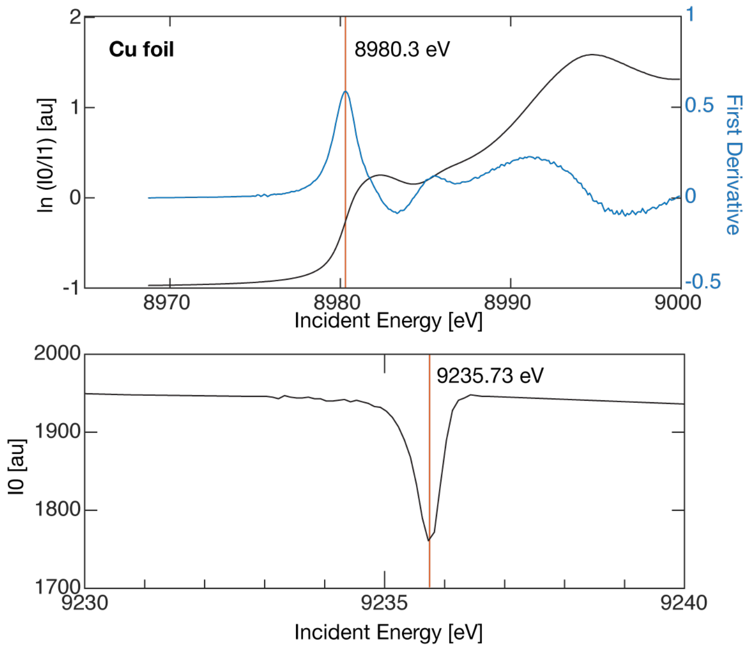

Figure S10. (Top) Monochromator calibration for BL6-2 measured by the transmission of a Cu foil, where the energy of the first inflection point is assumed to be 8980.3 eV. A sharp glitch in the Si(311) monochromator is observed at an energy of 9235.73 eV and was used for energy calibrations in the absence of the parallel foil scans.

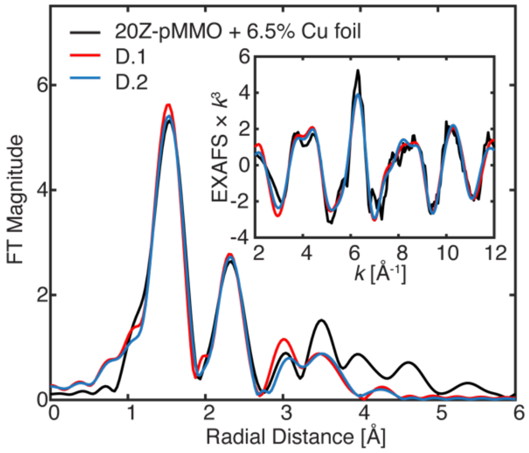

Figure S11. EXAFS fits of **20Z-pMMO-2018 + 6.5 Cu foil (6.5%)**.

Table S1. EXAFS fit parameters and statistics for **20Z-pMMO-2018 + 6.5% Cu Foil**

**Fit**	**Path**[a)]	***N***	***R* (Å)**	+/–	**σ^2 ($Å^2$)**	+/–	**ΔE_0**[b)]	χ[c)]
D.1	Cu-N/O (A)	3	1.970	0.010	0.00425	0.00075	-5.721	2.43
	Cu-C (B)	6	2.983	0.027	0.01095	0.00300		
	Cu-NN (C)	10	4.166	0.041	0.01162	0.00478		
	Cu-O (D)	2	2.563	0.013	0.00098	0.00130		
	Cu-C (E)	4	3.355	0.069	0.01876	0.01309		
D.2	Cu-N/O (A)	3	1.968	0.013	0.00419	0.00076	-6.151	2.43
	Cu-C (B)	6	2.959	0.081	0.02188	0.01019		
	Cu-NN (C)	10	4.160	0.045	0.01090	0.00446		
	Cu-O (D)	2	2.486	0.047	0.00360	0.00886		
	Cu-C (E)	4	3.421	0.114	0.02561	0.02433		
	Cu-Cu	0.5	2.566	0.024	0.00266	0.00393		

a) The scattering paths in parentheses correspond to those depicted in **Figure 9**.
b) The fitted ΔE_0 values for each are shifts relative to the set E_0 value of 8990.0.
c) The reduced χ^2 is normalized for the number of variables used in the fit.

Sample Larch Script for EXAFS Fitting

```
import numpy as np
from os import path as ospath

pwd = cwd()

# Open and process data from Athena project
pMMO_prj = read_athena('All_pMMO_XAS.prj')
exafsData = extract_athenagroup(pMMO_prj.PFY_high_conc_0_008flux_k12)

newplot(exafsData.xdat,exafsData.ydat,
      xlabel='Energy [eV]', ylabel = 'mu')

autobk(exafsData,rbkg = exafsData.bkg_params.rbkg,
     e0 = exafsData.bkg_params.e0,kw = exafsData.bkg_params.kw,
     kmax = exafsData.bkg_params.spl2,
     clamp_lo = exafsData.bkg_params.clamp1,
     clamp_hi = exafsData.bkg_params.clamp2)
      #takes Autobk parameters used in Athena

newplot(exafsData.xdat,exafsData.ydat)
plot(exafsData.xdat,exafsData.bkg)

newplot(exafsData.xdat,exafsData.flat)

exafsData.chi3 = exafsData.chi*exafsData.k**3

newplot(exafsData.k,exafsData.chi3,
      xlabel='k^3',
      ylabel='EXAFS x k^3')

xftf(exafsData.k, exafsData.chi, kmin=2, kmax=12, dk=1, window='hanning',
     kweight=3, group=exafsData)

newplot(exafsData.r,exafsData.chir_mag,
      xlabel='Radial Distance [Å]',
      ylabel='FT Magnitude',
      xmax = 6, xmin = 0, ymin = 0)

# Run feff of paths

feff6l(folder = pwd+'/feff_Cu_his/', feffinp = 'Cu_his.inp', verbose = True)
print('Done Feff1')

pars = group(amp  = param(0.9, vary=False),
             del_e0 = param(0.0, vary=True))
pathsToFit = []

# BEGINNING OF PATHS %%%%%%%%%%%%%%%%%%%%%%%%%%%%%%%%%%%%%%%%%%%

# path1 %%%%%%%%%%%%%%%%%%%%%%%%%%%%%%%%%%%%%%%%%%%%%%%%%%%%%%%%%

path1 = feffpath(pwd+'/feff_Cu_his/feff0001.dat',
               label = 'CuN',
               degen = 2.5,
               s02 = 'amp',
               e0= 'del_e0',
               sigma2 = 'sig2',
               deltar = 'del_r')
pars.sig2   = param(0.003, vary=True)
pars.del_r  = guess(0.0, vary=True)
pathsToFit.append(path1)

# END OF PATHS %%%%%%%%%%%%%%%%%%%%%%%%%%%%%%%%%%%%%%%%%%%%%%%%%%%

# FITTING %%%%%%%%%%%%%%%%%%%%%%%%%%%%%%%%%%%%%%%%%%%%%%%%%%%%%%%%

trans = feffit_transform(kmin=2, kmax=12, kw=3, dk=1, window='hanning', rmin=1, rmax=4)
dset = feffit_dataset(data=exafsData, pathlist=pathsToFit,transform=trans)
out = feffit(paramgroup = pars, datasets = dset)

print(feffit_report(out))

# OUTPUT %%%%%%%%%%%%%%%%%%%%%%%%%%%%%%%%%%%%%%%%%%%%%%%%%%%%%%%%%

fitNum = 1 # either manually type or initialize and use fitNum+1
```

```
fileOut = 'fits/fit'+str(fitNum).zfill(3)

if (ospath.isfile(fileOut+'.out') == True):
      print('FILE ALREADY EXISTS')
      exit()
else:
      try:
        fout = open(pwd+'/' +fileOut + '.out', 'w')
        fout.write("%s\n" % feffit_report(out))
        fout.close()
      except:
        print('could not write doc_feffit1.out')
      endtry

      cd(pwd)

      print('Finished pMMO EXAFS')

      # plot fitted R
      newplot(dset.data.r,dset.data.chir_mag,xmin = 0, xmax = 6, ymin = 0,label='Data',
        xlabel = 'Radial Distance [Å]', ylabel = 'FT Magnitude', title = dset.data.label)

      plot(dset.model.r,dset.model.chir_mag,label='Fit')
      save_plot(fileOut + '_r.eps', format = 'eps', transparent = True)

      # plot fitted k
      newplot(dset.data.k,dset.data.chi*dset.data.k**3,xmin = 2, xmax = 12,label='Data',
        xlabel  =  r'$k$  $\rm(\AA^{-1})  $',  ylabel  =  r'EXAFS  $\times$  $k^{3}  $',  title  =
      dset.data.label)

      plot(dset.model.k,dset.model.chi*dset.model.k**3,label='Fit')
      save_plot(fileOut + '_k.eps', format = 'eps', transparent = True)

      newplot(dset.data.r,dset.data.chir_mag, label='Data',
        xlabel = r'$k$ $\rm(\AA^{-1}) $', ylabel = r'EXAFS $\times$ $k^{3} $',
         title = dset.data.label, win = 1,xmin = 0, xmax = 6)
      plot_paths_r(dset, offset=-0.5, rmax=None, show_mag=True, show_real=False,
                    show_imag=False, new=False, win = 1)
      redraw_plot(win = 1)
      save_plot(fileOut + '_r_stacked.eps', format = 'eps', transparent = True)

      newplot(dset.data.k,dset.data.chi*dset.data.k**3, label='Data',
        xlabel = r'$k$ $\rm(\AA^{-1}) $', ylabel = r'EXAFS $\times$ $k^{3} $',
         title = dset.data.label, win =2,xmin = 2, xmax = 12)
      plot_paths_k(dset, offset=-2, rmax=None, show_mag=True, show_real=False,
                    show_imag=False, new=False, win = 2)
      redraw_plot(win = 2)

      save_plot(fileOut + '_k_stacked.eps', format = 'eps', transparent = True, win  = 2)

      outR = []
      outR.append((pathsToFit[0].r))
      outR.append(dset.data.chir_mag)
      outR.append(dset.model.chir_mag)
      for each in pathsToFit:
        outR.append((each.chir_mag))
      endfor
      np.savetxt(fileOut+'_indpaths_r.txt',np.transpose(outR),'%.8f')

      outK = []
      outK.append((pathsToFit[0].k))
      outK.append(dset.data.chi*dset.data.k**3)
      outK.append(dset.model.chi*dset.data.k**3)
      for each in pathsToFit:
        outK.append((each.chi*each.k**3))
      endfor
      np.savetxt(fileOut+'_indpaths_k.txt',np.transpose(outK),'%.8f')
endif
```

References

1. M. O. Ross, F. MacMillan, J. Wang, A. Nisthal, T. J. Lawton, B. D. Olafson, S. L. Mayo, A. C. Rosenzweig and B. M. Hoffman, *Science*, 2019, **364**, 566-570.
2. S. Y. Ro, M. O. Ross, Y. W. Deng, S. Batelu, T. J. Lawton, J. D. Hurley, T. L. Stemmler, B. M. Hoffman and A. C. Rosenzweig, *J. Biol. Chem.*, 2018, **293**, 10457-10465.
3. K. L. Summers, K. M. Schilling, G. Roseman, K. A. Markham, N. V. Dolgova, T. Kroll, D. Sokaras, G. L. Millhauser, I. J. Pickering and G. N. George, *Inorg. Chem.*, 2019, **58**, 6294-6311.
4. R. L. Lieberman, K. C. Kondapalli, D. B. Shrestha, A. S. Hakemian, S. M. Smith, J. Telser, J. Kuzelka, R. Gupta, A. S. Borovik, S. J. Lippard, B. M. Hoffman, A. C. Rosenzweig and T. L. Stemmler, *Inorg. Chem.*, 2006, **45**, 8372-8381.
5. S. Y. Ro, L. F. Schachner, C. W. Koo, R. Purohit, J. P. Remis, G. E. Kenney, B. W. Liauw, P. M. Thomas, S. M. Patrie, N. L. Kelleher and A. C. Rosenzweig, *Nat. Commun.*, 2019, **10**, 2675.
